# Rare variable *M*. *tuberculosis* antigens induce predominant Th17 responses in human infection

**DOI:** 10.1172/jci.insight.202134

**Published:** 2026-01-27

**Authors:** Paul Ogongo, Liya Wassie, Anthony Tran, Devin Columbus, Julia Huffaker, Lisa Sharling, Gregory Ouma, Samuel Gurrion Ouma, Kidist Bobosha, Cecilia S. Lindestam Arlehamn, Neel R. Gandhi, Sara C. Auld, Jyothi Rengarajan, Cheryl L. Day, Artur Queiroz, Mariana Araújo-Pereira, Eduardo Fukutani, Bruno B. Andrade, John D. Altman, Henry M. Blumberg, Joel D. Ernst, the TBRU ASTRa Study Group

**Affiliations:** 1 Division of Experimental Medicine, UCSF, San Francisco, California, USA.; 2 Department of Tropical and Infectious Diseases, Kenya Institute of Primate Research, Nairobi, Kenya.; 3 Mycobacterial Disease Research Directorate, Armauer Hansen Research Institute, Addis Ababa, Ethiopia.; 4 Department of Epidemiology, Emory University Rollins School of Public Health, Atlanta, Georgia, USA.; 5 Center for Global Health Research, Kenya Medical Research Institute, Kisumu, Kenya.; 6 Center for Vaccine Innovation, La Jolla Institute for Immunology, La Jolla, California, USA.; 7 Department of Infectious Disease and Immunology, Center for Vaccine Research, Statens Serum Institut, Copenhagen, Denmark.; 8 Department of Medicine, Division of Infectious Diseases, Emory University School of Medicine, Atlanta, Georgia, USA.; 9 Department of Global Health, Emory University Rollins School of Public Health, Atlanta, Georgia, USA.; 10 Department of Medicine, Division of Pulmonary and Critical Care Medicine, Emory University School of Medicine, Atlanta, Georgia, USA.; 11 Emory Vaccine Center, Emory University, Atlanta, Georgia, USA.; 12 Department of Microbiology and Immunology, Emory University School of Medicine, Atlanta, Georgia, USA.; 13 Multinational Organization Network Sponsoring Translational and Epidemiological Research (MONSTER) Initiative, Salvador, Brazil.; 14 Laboratório de Pesquisa Clínica e Translacional, Instituto Gonçalo Moniz, Fundação Oswaldo Cruz, Salvador, Brazil.; 15 TBRU ASTRa Study Group is detailed in Supplemental Acknowledgments.

**Keywords:** Immunology, Infectious disease, Microbiology, Antigen, T cells, Tuberculosis

## Abstract

CD4 T cells are essential for immunity to M *tuberculosis* (*Mtb*), and emerging evidence indicates that IL-17–producing Th17 cells contribute to immunity to *Mtb*. While identifying protective T cell effector functions is important for TB vaccine design, T cell antigen specificity is also likely to be important. To identify antigens that induce protective immunity, we reasoned that, as in other pathogens, effective immune recognition drives sequence diversity in individual *Mtb* antigens. We previously identified *Mtb* genes under evolutionary diversifying selection pressure whose products we term Rare Variable *Mtb* Antigens (RVMA). Here, in 2 distinct human cohorts with recent exposure to TB, we found that RVMA preferentially induce CD4 T cells that express RoRγt and produce IL-17, in contrast to “classical” *Mtb* antigens that induce T cells that produce IFN-γ. Together with emerging evidence showing human Th17 responses are associated with prevention of progression to TB disease, our results suggest that RVMA can be valuable antigens in vaccines for those already infected with *Mtb* to amplify existing antigen-specific Th17 responses to prevent TB disease.

## Abstract 2

Different individual Mycobacterium tuberculosis antigens induce distinct T cell responses, with important implications for TB vaccine development.

## Graphical abstract

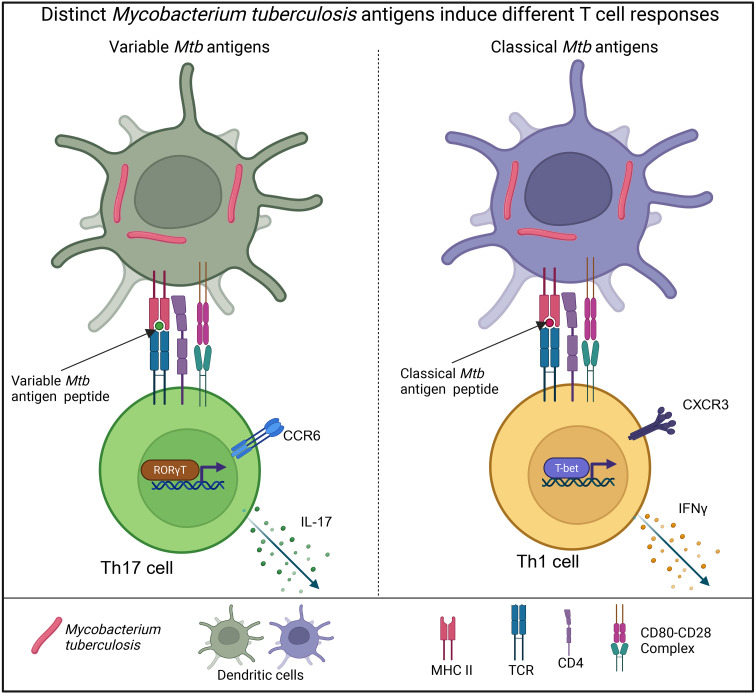

## Introduction

After infection with *Mycobacterium tuberculosis* (*Mtb*), the majority of individuals remain well and do not develop active tuberculosis (TB) disease, suggesting that some human immune responses can control the infection. Evidence from people with HIV (1–3) and animal models (4–7) has revealed that CD4 T cells are essential for immune control of *Mtb*, but the properties of CD4 T cells that contribute to immune control have not been fully defined. While considerable effort has been focused on identifying T cell effector functions associated with protection, less attention has been given to antigen specificity of T cells as a determinant of immune control, despite the *Mtb* genome encoding ~4,000 potentially antigenic proteins (8).

Antigen and epitope specificity are critical determinants of protective immunity in other infectious diseases. Antibodies to pathogens such as Dengue virus (DV) (9, 10), respiratory syncytial virus (RSV) (11, 12), and *Neisseria meningitidis* (13) can be associated with protection or not, or even with more severe disease, depending on the antigen and epitope specificity. For infectious diseases in which T cell responses play predominant roles in protection, there is less knowledge of the importance of distinct antigen targets. However, in HIV, there is evidence that CD4 and CD8 T cells that recognize the env protein are associated with poorer control, while T cells that recognize gag are associated with better control of viremia (14, 15). A recent report emphasized the relevance of identifying distinct *Mtb* antigen–specific T cell repertoires, as it identified T cell antigen receptor (TCR) clonotypes that are associated with control (maintained latent TB) or progression to active TB disease (16).

A characteristic feature of host-pathogen relationships is the coevolutionary arms race in which antigens that induce host-protective (and therefore pathogen-detrimental) immune responses are driven to escape recognition through diversifying evolutionary selection and antigenic variation. Antigenic variation to escape immune recognition has been observed for diverse pathogens, including viruses like HIV (17), HCV (18), and influenza (19); bacteria, including *Streptococcus pneumoniae* (20) and *Neisseria gonorrhoeae* (21); parasites such as *Trypanosoma brucei* (22) and *Plasmodium falciparum* (23, 24); and even tumor cells (25–28). In an earlier study, we hypothesized that antigenic targets of protective immunity to *Mtb* could be discovered by studying evolutionary selection pressure through T cell epitope sequence variation in phylogeographically diverse clinical isolates. We made the unexpected discovery that the great majority of experimentally verified human T cell epitopes in *Mtb* were perfectly conserved and exhibited no evidence of antigenic variation (29). This result raised the question of whether there were undiscovered antigens and epitopes under diversifying selection pressure from human T cell recognition. In a subsequent study, we used comparative genomics to discover *Mtb* genes that exhibit evidence of the strongest diversifying evolutionary selection pressure. We determined that the proteins encoded by those genes are antigenic and that the observed amino acid substitutions affect human T cell responses (30). We termed these antigenic proteins the Rare Variable *Mtb* Antigens (RVMA).

In the present study, we tested the hypothesis that RVMA express functions distinct from those that recognize classical *Mtb* antigens (characterized by robust Th1 cell responses) (31–36). We first studied 60 distinct *Mtb* antigens (37) using whole blood ELISA, and we found that the classical *Mtb* antigens elicit higher prevalence (percentage of samples with a measurable response) and magnitude (amount of cytokine, or percentage of T cells responding) IFN-γ responses compared with other *Mtb* antigens, including RVMA. To comprehensively analyze distinct *Mtb* antigen responses, we stimulated peripheral blood mononuclear cells (PBMC) from the same donors and assessed cytokine (TNF-α, IFN-γ, IL-17, and GM-CSF) production by CD4 T cells using intracellular cytokine staining (ICS). Depending on the number of PBMC available in a given sample, we selected up to 4 RVMA (Rv0010c, Rv0012, RimJ, and LldD2) and 4 classical antigens (with the highest IFN-γ response as measured by ELISA from the 60-antigen set) for each assay. We found that CD4 T cells that recognize RVMA predominantly produce IL-17 and express RORγT, while confirming that classical *Mtb* antigens drive Th1 responses characterized by high IFN-γ production and high expression of T-bet and CXCR3. In additional experiments, we evaluated the remaining 3 RVMA antigens (Rv0990c, Rv2719c, and Rv3221c) along with 4 conserved antigens that previously showed low IFN-γ responses by ELISA (Rv1366, Rv1957, Rv2022c, and Rv2031c), collectively termed low IFN-γ conserved antigens (LICA). The results confirmed our earlier findings: RVMA antigens elicited stronger IL-17 responses compared with conserved antigens, consistent with a Th17-biased immune profile. Moreover, ICS results aligned with ELISA data, verifying that Rv1366, Rv1957, Rv2022c, and Rv2031c induced lower IFN-γ responses. These findings reveal previously unknown skewing of Th17 responses to the rare *Mtb* antigens that are characterized by diversifying evolutionary selection pressure and confirm heterogeneity in IFN-γ responses to distinct *Mtb* antigens.

## Results

### Individual Mtb antigens elicit IFN-γ responses that vary in frequency and magnitude

**Figure 1:**
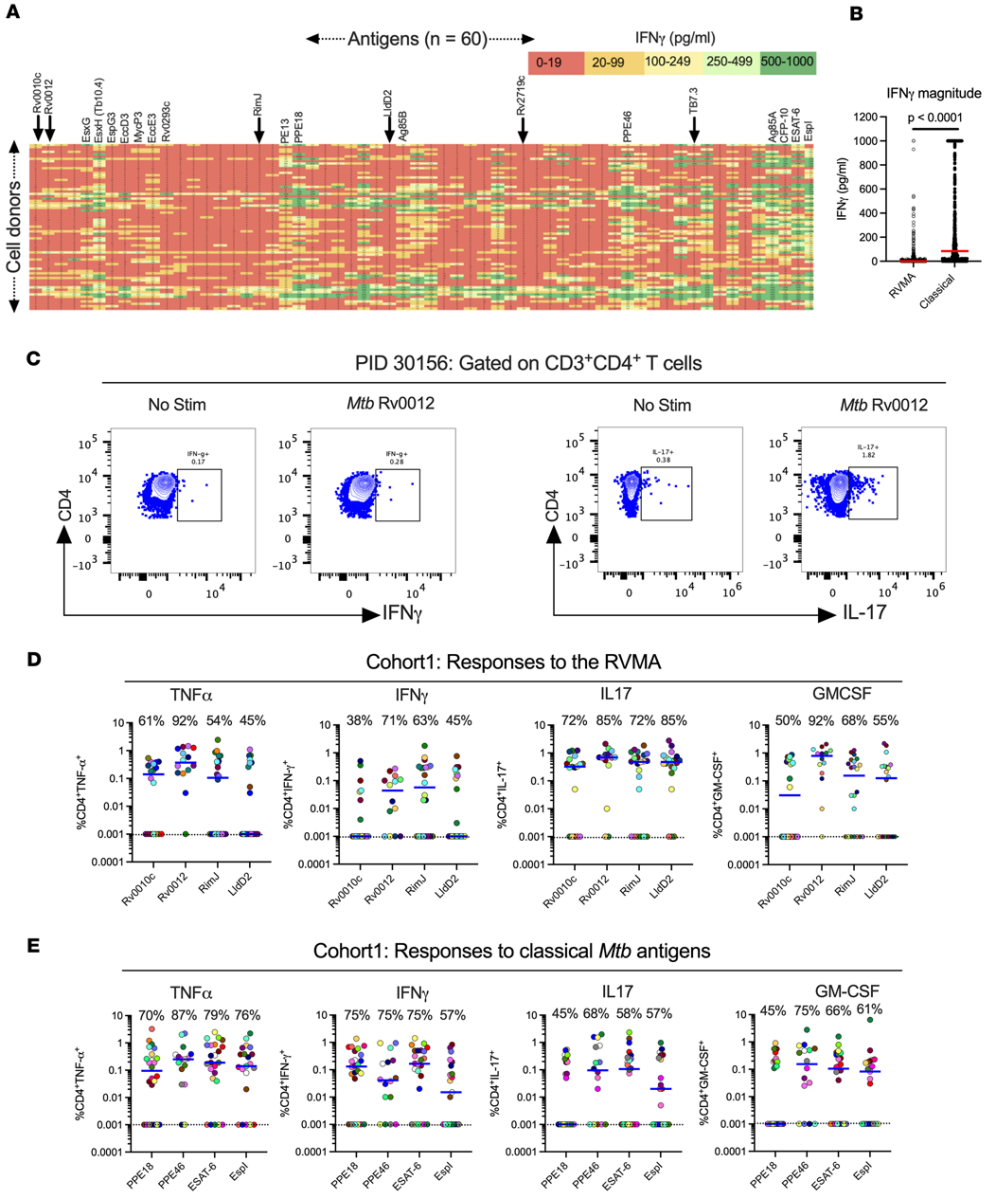
Distinct *Mtb* antigens elicit T cell responses with different functional properties (Cohort 1). (**A**) Whole blood samples from QFT^+^HIV^–^ participants in Cohort 1 (KEMRI, Kisumu, Kenya) were stimulated with 60 individual *Mtb* antigens as overlapping peptides (1 μg/mL) for 7 days, and supernatants were harvested to quantitate IFN-γ by ELISA. The antigens are arranged left to right (antigens 1–60) according to their positions on the *Mtb* chromosome, and individual participant samples are arranged in rows. Data presented are results after subtraction of the average of 6 unstimulated wells as the background response. IFN-γ levels are color coded according to magnitude, with red for the lowest and green for the highest responses. Responses greater than the highest standard were assigned the value of the highest standard (1,000 pg/mL). Selected antigens are highlighted at the top of the heatmap, with arrows indicating 6 of the 7 RVMA. The identity of all of the antigens ordered in the same fashion is available in ref. 37. (**B**) Whole blood IFN-γ response magnitude of selected classical antigens (Tb10.4 [EsxH], PE13, PPE18, Ag85B, PPE46, Ag85A, CFP-10, ESAT-6, and EspI) compared with the 6 RVMA (Rv0010c, Rv0012, RimJ, LldD2, Rv2719c, and TB7.3); the red horizontal line represents the median response. Statistical significance was determined by the Mann-Whitney *U* test. (**C**) Representative flow cytometry plots comparing CD4 T cells producing IFN-γ or IL-17 in response to stimulation with one of the RVMA (Rv0012); background response for each cytokine is shown for comparison. (**D** and **E**) Cryopreserved peripheral blood mononuclear cells (PBMC) were stimulated with indicated antigens (2 mg/mL) for a total of 20 hours in the presence of Golgi Stop and Golgi Plug as well as costimulatory antibodies anti-CD28 and anti-CD49d; cytokine production by CD4^+^ T cells was determined by intracellular cytokine staining and flow cytometry. Data are values after subtraction of unstimulated cells, with values lower than the unstimulated control cells indicated below the dotted line (cut-off of positive response). The frequency of participants with a detectable response is shown at the top of each antigen plot. Each symbol is a distinct participant; the horizontal blue line indicates the median cytokine response. Results for RVMA are shown in **D**, and results for the classical antigens are shown in **E**.

To characterize the frequency (the percentage of participants whose cells responded) and magnitude (the quantitative response in a given sample) of responses to 60 individual *Mtb* antigens, we assayed samples from 71 QFT^+^HIV^–^ adults after recent (≤3 months) household exposure to active pulmonary TB from western Kenya (Cohort 1). After quality control and subtraction of background, we found considerable diversity in IFN-γ secretion by antigen (columns) and by participant (rows) (Figure 1A). By arranging the antigens by the position of their genes on the *Mtb* chromosome, the results reveal apparent “antigenic islands” and “antigenic deserts” reflected in the frequency (percentage of samples with responses) and magnitude (amount of IFN-γ produced) of responses. The antigenic islands largely comprise known *Mtb* classical antigens, including EsxH (Tb10.4), PE13, PPE18, Ag85B, PPE46, Ag85A, CFP-10, and ESAT-6 (median values, 50–279 pg/mL), consistent with other reports that these antigens induce IFN-γ responses. In some samples, responses to these antigens exceeded the upper limit of the IFN-γ assay (1,000 pg/mL), contributing to potential underestimation of the response magnitudes.

In contrast to the findings in ref. 33, our assay platform and participant population did not show evidence for an antigenic island involving the ESX-3 locus. While EsxH (also known as Tb10.4) induced responses of modest magnitudes (median, 102 pg/mL), the other components of the Esx-3 locus (EsxG, EspG3, EccD3, MycP3, EccE3, and Rv0293c) induced minimal IFN-γ responses (medians, 4–14 pg/mL).

Compared with responses to the known classical antigens, we found lower magnitude IFN-γ responses (0–19 pg/mL; red color) to other *Mtb* antigens. The antigens that induced low magnitude IFN-γ responses include Rv0010c, Rv0012, RimJ, LldD2, Rv2719c, and TB7.3 (Rv3221c), which we previously identified as showing evidence of evolutionary diversifying selection (shown in arrows) and termed RVMA (30). While the overall IFN-γ responses to RVMA were low, some participants had higher magnitude IFN-γ responses (>100 pg/mL) to certain RVMA, although these were still lower than the magnitude of responses to the classical antigens (Figure 1B).

We also observed variation in responses to each of the 60 antigens at the level of individual participants (Figure 1A, rows), emphasizing that responses to a single *Mtb* antigen are not representative of the response to other *Mtb* antigens within an individual. Thus, we confirmed the diversity in responses to *Mtb* infection in humans in agreement with a previous study (38) and found that classical *Mtb* antigens elicit a robust IFN-γ response in whole blood of individuals with LTBI, while other antigens, including the RVMA, elicit lower frequency and lower magnitude IFN-γ responses.

### Mtb antigens induce diverse cytokine responses that vary by antigen class

#### Response frequencies.

Since RVMA are characterized by evidence of diversifying evolutionary selection and T cell epitope sequence variation, we hypothesized that they induce CD4 T cell responses distinct from those induced by the commonly studied classical *Mtb* antigens. We therefore asked whether RVMA induce CD4 T cells that express cytokines other than IFN-γ. For this analysis (and guided by the cell input requirement), we first randomly selected 4 RVMA that induced low frequency and low magnitude IFN-γ responses in the 60-antigen whole blood assay and compared the responses to 4 classical *Mtb* antigens that induced high frequency and high-magnitude IFN-γ responses. We stimulated PBMC from participants in Cohort 1 (Supplemental Table 1; supplemental material available online with this article; https://doi.org/10.1172/jci.insight.202134DS1) with the individual antigens and quantitated CD4 T cells expressing TNF-α, IFN-γ, IL-17, and/or GM-CSF by ICS and flow cytometry (Figure 1C and Supplemental Figure 1).

Consistent with the results of the whole blood assay, the 4 RVMA (Rv0010c, Rv0012, RimJ, and LldD2) induced IFN-γ–producing CD4 T cells in only a modest fraction of participants (38%–71%, depending on the individual antigen). In contrast, CD4 T cells responding to individual RVMA produced IL-17 in a high proportion (72%–85%) of participants. The frequency of participants whose CD4 T cells produced TNF-α or GM-CSF in response to the RVMA varied widely (45%–92%, depending on the individual antigen) (Figure 1D and Table 1).

At the level of individual antigens, all 4 RVMA induced IL-17–producing CD4 T cells in > 70% of the participants, while other cytokine responses varied widely by antigen. Of the RVMA, Rv0010c induced IFN-γ and GM-CSF at the lowest frequency (38% and 50%, respectively). Notably, Rv0012 induced GM-CSF–producing CD4 T cells in a high frequency (92%) of participants, a rate higher than any of the other RVMA or any of the classical antigens. RimJ induced TNF-α in only 54% and induced IFN-γ and GM-CSF with frequencies intermediate between those of IL-17 and TNF-α. LldD2 induced the lowest frequencies of TNF-α–, IFN-γ–, or GM-CSF–producing CD4 T cells (45%, 45%, and 55%, respectively) but induced IL-17–producing cells in 85% (Figure 1D and Table 1).

For comparison, we studied CD4 T cell production of the same 4 cytokines in response to 4 IFN-γ–dominant classical antigens. This revealed considerable variation in the frequency of individuals who responded with CD4 T cell production of TNF-α, IFN-γ, IL-17, and GM-CSF after stimulation with individual classical antigens (PPE18 [Rv1196], PPE46 [Rv3018c], ESAT-6 [Rv3875], and EspI [Rv3876]) (Figure 1E and Table 1). TNF-α responses to individual classical antigens were observed in 70%–87% of samples, with the highest frequency of TNF-α responses to PPE46. IFN-γ responses to the 4 classical antigens were observed in 57%–75% of samples, with little variation in the frequency of responses to PPE18, PPE46, and ESAT-6 and fewer responders to EspI. By comparison, the 4 classical antigens induced IL-17 (45%–68%) and GM-CSF (45%–75%) depending on the antigen.

When we compared the frequency of responders (that is, participants whose CD4 T cells expressed a given cytokine in response to *Mtb* antigen stimulation) by antigen class (i.e., classical versus RVMA), IL-17 responses were more frequent for RVMA than for classical *Mtb* antigens, while TNF-α, IFN-γ, and GM-CSF responses did not differ significantly (Table 2).

These data indicate that *Mtb* antigens vary in their induction of CD4 T cells that express different cytokines. The data also demonstrate that RVMA induce IL-17–producing CD4 T cells more frequently than IFN-γ– or TNF-α–producing T cells, and they induce IL-17 responses more frequently than do the classical antigens in a cohort of recently exposed household contacts (HHCs) that have controlled *Mtb* infection.

#### Response magnitudes.

In addition to variation in the proportion (frequency) of participants whose CD4 T cells responded to individual antigens with expression of different cytokines, we found variation in the magnitude of the CD4 T cell responses, defined as the percent of CD4 T cells that produced the cytokine of interest in response to individual antigens.

For each of the individual RVMA, the magnitude of IL-17 responses was greater than for TNF-α, IFN-γ, or GM-CSF, with the exception that Rv0012 induced GM-CSF responses that exceeded those of each of the other 3 cytokines (Figure 1D and Table 3). As expected for the individual classical antigens, the magnitudes of TNF-α and IFN-γ response magnitudes were highest, with the exception that PPE46 induced GM-CSF responses that exceeded the magnitude of IFN-γ responses to Espl. IL-17 responses were present with the lowest magnitudes for all 4 of the classical antigens.

For the RVMA as a group, the highest magnitude responses were for CD4 T cells that produce IL-17 (median, 0.46% of CD4 T cells), while the lowest magnitude responses were for IFN-γ (median 0.01%) (Figure 1D and Table 4). GM-CSF– and TNF-α–producing CD4 T cells were found with intermediate magnitudes (medians of 0.20% and 0.15%, respectively). In contrast, for the classical antigens as a group, the highest magnitude responses were for TNF-α–producing CD4 T cells (median, 0.19% of CD4 T cells) with slightly lower magnitude responses for IFN-γ (0.08%), IL-17 (0.05%), and GM-CSF (0.09%). When comparing cytokine responses by antigen group, differences were significant for IL-17 (RVMA > classical) and IFN-γ (classical > RVMA) (Table 4). These analyses revealed that, consistent with the frequency of responders, the magnitude of IL-17–producing CD4 T cells is higher for RVMA than for classical antigens.

To determine whether the IL-17 bias of CD4 T cell responses to RVMA extends to another human population, we used the same experimental procedures to study samples from participants recruited in the same manner and with the same criteria as for Cohort 1, in an independent cohort in Addis Ababa, Ethiopia (Cohort 2). The 2 cohorts were comparable in their distribution of age, sex, body mass index (BMI), and hemoglobin A1c (Supplemental Table 1). The distribution of sex was comparable between the cohorts (*P* = 0.2611, Fisher’s exact test), and when we considered sex as a biological variable, we found no systematic differences in the results within or between cohorts according to sex. Results of QuantiFERON testing differed between the cohorts: Cohort 1 had significantly higher magnitude responses for TB Antigen minus Nil, while Cohort 2 had higher responses for Mitogen minus Nil (Supplemental Table 1).

**Figure 2:**
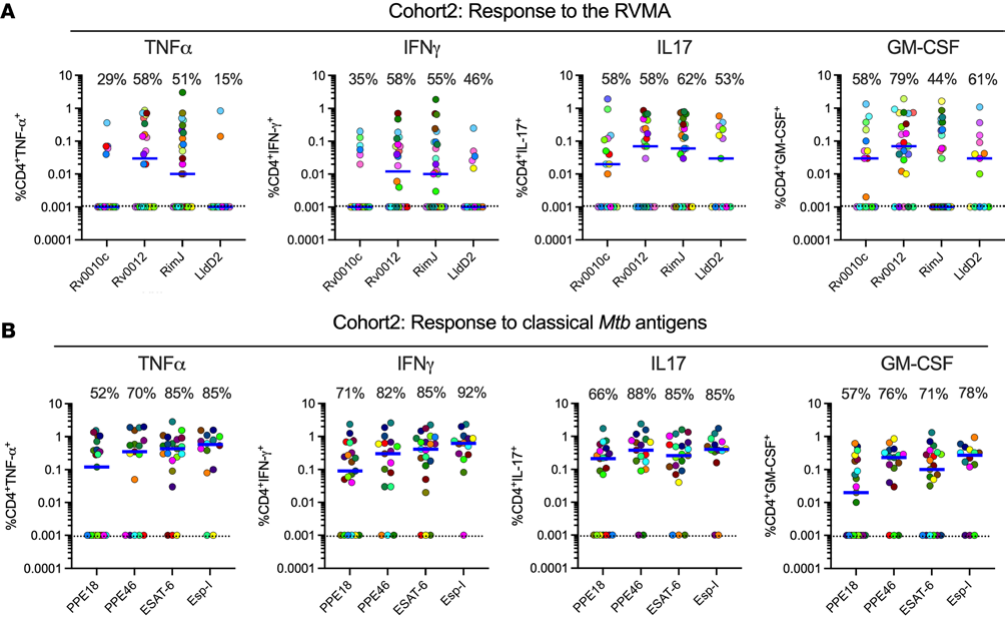
Distinct *Mtb* antigens elicit T cell responses with different functional properties (Cohort 2). Procedures and analyses were as described in Figure 1; the samples were obtained from participants in Cohort 2 (AHRI, Addis Ababa, Ethiopia). (**A** and **B**) Results for RVMA are shown in **A**; results for classical *Mtb* antigens are shown in **B**.

In Cohort 2, the frequency of participants whose cells responded to RVMA was lower than for classical antigens; the difference was significant for IFN-γ and for IL-17 but not for TNF-α or GM-CSF (Figure 2, A and B, and Supplemental Tables 2 and 3). Likewise, the magnitudes of cytokine-producing CD4 T cells in Cohort 2 were lower in response to RVMA compared with classical antigens; the differences in magnitudes between RVMA and classical antigens were significant for TNF-α, IFN-γ, and IL-17 but not for GM-CSF (Supplemental Table 4). Because of the lower-magnitude responses, the IL-17 response magnitudes to the RVMA did not exceed the IL-17 responses to classical antigens. Despite the overall lower-magnitude responses, IL-17 responses were higher than other cytokine responses to RVMA (Figure 2A and Supplemental Table 4). When we analyzed IL-17 and IFN-γ responses to individual RVMA in Cohort 2, we found that the frequencies of IL-17 responders exceeded those for IFN-γ for all 4 of the RVMA (Supplemental Table 5). Likewise, the magnitudes of IL-17 responses exceeded those of IFN-γ responses for all 4 of the RVMA; the differences were significant for Rv0012 and LldD2. Comparison of the cytokine response magnitudes to all of the RVMA together revealed that IL-17 responses were significantly greater than TNF-α or IFN-γ (*P* = 0.0378 and 0.0018, respectively) but not GM-CSF (Friedman test with Dunn’s correction for multiple comparisons). Despite the differences in the overall magnitudes of CD4 T cell responses to RVMA in Cohort 2 compared with Cohort 1, the results confirmed the findings in Cohort 1 that RVMA predominantly induce CD4 T cells that produce IL-17.

In additional experiments, we assessed the same cytokine responses to the remaining 3 RVMA (Rv0990c, Rv2719c, and Rv3221c) (30), and 4 additional antigens that are conserved and nonsecreted proteins and that exhibited low IFN-γ responses by ELISA (Rv1366, Rv1957, Rv2022c, and Rv2031c) — the LICA — using PBMC obtained from Cohort 2 participants. Consistent with findings from the first RVMA set, these antigens predominantly induced IL-17 responses, both in response frequencies and magnitudes (Supplemental Figure 2 and Supplemental Table 5), with the hierarchy of responses being IL-17 > GM-CSF > IFN-γ > TNF-α. IL-17 responses were significantly greater than TNF-α or IFN-γ (*P* = 0.0005 and 0.0478, respectively) but not GM-CSF (Friedman test with Dunn’s correction for multiple comparisons). Rv3221c elicited the highest frequency and magnitude responses (57%–87%; 0.04%–0.51% of CD4 T cells) for any of the cytokines (Supplemental Figure 2 and Supplemental Table 5). Grouped analysis of RVMA revealed significantly higher responses for IL-17 than IFN-γ (Supplemental Table 6).

The LICA induced strong IL-17 and GM-CSF responses (>71% prevalence for each antigen) with comparable magnitudes across antigens (Supplemental Figure 2). IFN-γ responses were lower (54%–71% prevalence; median magnitude 0.04%–0.19 % of CD4 T cells), whereas TNF-α responses were the lowest — e.g., Rv1366 responses were detectable in only 21% of donors with a median magnitude below the limit of detection. When grouped, the LICA showed significantly higher IL-17 than IFN-γ responses (Supplemental Table 6). Collectively, these findings reinforce the preferential induction of IL-17 by RVMA and underscore heterogeneity in IFN-γ responses to classical *Mtb* antigens.

In Cohort 1, RVMA and IFN-γ–dominant classical antigens induced similar magnitudes of TNF^+^IL-17^+^ and IFN-γ^+^IL-17^+^ cells, while RVMA induced lower magnitudes of TNF^+^IFN-γ^+^ cells than did classical antigens. In Cohort 2, RVMA induced lower magnitudes of cells expressing each of the 3 dual cytokine combinations compared with those induced by IFN-γ–dominant classical antigens (Supplemental Figure 3).

### RVMA-responsive CD4 T cells include bona fide Th17 cells

**Figure 3:**
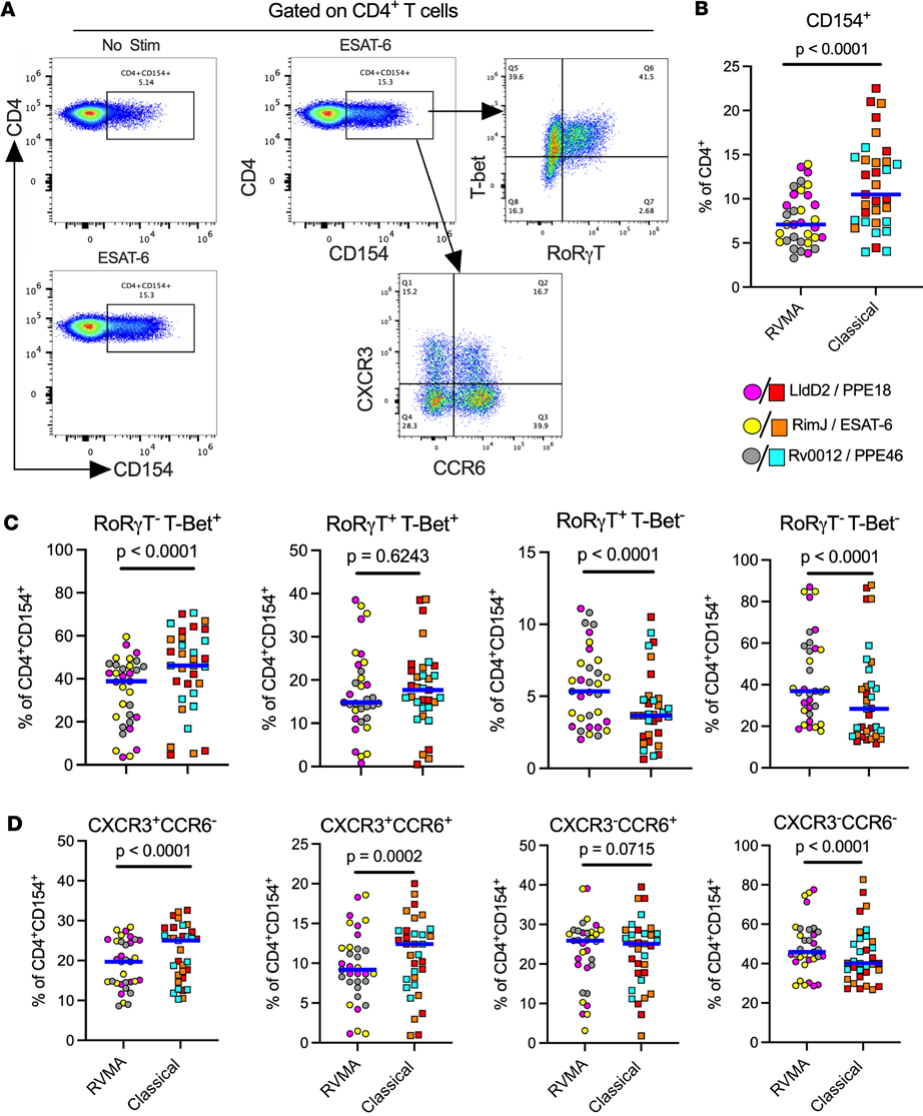
Antigen-activated cells express markers of Th17 and Th1 cells upon stimulation with RVMA and classical antigens, respectively. Cryopreserved PBMC from participants in Cohort 1 were stimulated with antigens for a total of 20 hours in the presence of costimulatory antibodies anti-CD28 and anti-CD49d and anti-CD40 blocking antibody without protein transport inhibitors, and antigen-activated cells were identified by CD154 surface expression on CD4^+^ T cells. (**A**) Representative flow cytometry plots. (**B**) CD154 surface expression on CD4^+^ T cells; CD154^+^ was used to identify antigen-activated T cells. (**C** and **D**) Expression of Th cell lineage-defining transcription factors (RORγT = Th17 and T-bet = Th1) (**C**) and expression of chemokine receptors (CCR6 = Th17 marker) and (CXCR3 = Th1 marker) on antigen-activated T cells (**D**). Wilcoxon matched pairs test was used.

To further investigate CD4 T cells that respond to RVMA, we extended our studies to include additional markers associated with Th17 cells. Since peptide:HLA multimers for the Cohort 1 and Cohort 2 participants’ HLA alleles are not yet available, we used a T cell activation induced marker (AIM) assay (39–41) based on surface expression of CD154 after brief antigen stimulation in the absence of protein transport inhibitors, and we then assayed expression of RORγT and CCR6 (Th17) or T-bet and CXCR3 (Th1) on the *Mtb* antigen–activated CD154^+^ cells (Figure 3A). Stimulation with RVMA induced lower frequencies of CD154^+^ CD4 T cells in PBMC than did classical antigens (Figure 3B), consistent with immunodominance of classical antigens in the latter group. When we analyzed lineage-defining transcription factor (TF) expression on antigen-responsive (CD4^+^CD154^+^) cells, we found a significantly higher fraction of RORγT^+^T-bet^–^ cells in RVMA-responsive cells than in cells that responded to classical antigens (Figure 3C). In turn, we observed a significantly higher fraction of RORγT^–^T-bet^+^ cells responding to classical antigens than to RVMA. These results are in accord with the cytokine data (Figures 1 and 2) and indicate that, beyond cytokine production, RVMA-responsive CD4 T cells exhibit other characteristics of Th17 cells while confirming that classical antigen-responsive T cells are typical Th1 cells. We found no difference in double-positive (RORγT^+^T-bet^+^) cells according to antigen category (Figure 3C).

Since certain chemokine receptors (CRs) are reported to be associated with specific CD4 T cell subsets, we also examined the expression of CCR6 (characteristic of Th17 cells) and CXCR3 (characteristic of Th1 cells). This revealed a significantly higher incidence of CXCR3^+^CCR6^–^ (Th1) and CXCR3^+^CCR6^+^ (Th1*) on antigen-activated (CD4^+^CD154^+^) cells responding to classical antigens than to the RVMA (Figure 3D), consistent with the previously reported *Mtb*-responsive human Th1* responses to *Mtb* peptide pools (33, 42–44). In contrast, the incidence of CD4^+^CD154^+^ cells that were CXCR3^–^CCR6^+^ (Figure 3D) did not differ by antigen class. Notably, T cells that responded (CD4^+^CD154^+^) to RVMA exhibit a higher incidence of being CXCR3^–^CCR6^–^ than are classical antigen-activated CD4^+^ cells, suggesting that some RVMA-responsive cells do not adhere to a conventional pattern of CR expression.

### RVMA are enriched for nonclassical Th1 or Th17 responses

The ICS data reveal prevalent detection of GM-CSF (50%–92%, depending on antigen, with Rv0012 particularly robust in inducing GM-CSF production) in both Cohort 1 and Cohort 2 (Figure 1D, Figure 2A, and Supplemental Figure 2A), with a relative magnitude of 0.1% of CD4^+^ T cells. The cytokine GM-CSF has been little studied in TB, and its role in the outcome of *Mtb* infection remains unclear, although studies in mice indicate that T cell–derived GM-CSF can contribute to restricting infection (45). We also observed enrichment of CD154^+^CCR6^–^CXCR3^–^ and CD154^+^T-bet^–^RORγT^–^ RVMA-responsive CD4 T cells compared with classical antigens (Figure 3). To comprehensively investigate whether RVMA induce T cell responses with phenotypes other than classical Th17 (and in some cases low-level Th1), we selected participants in whom cytokines, TFs, and CRs were measured and antigens for which this data was complete. This yielded 41 samples in Cohort 1 and 29 samples in Cohort 2 with 3 RVMA (Rv0012, RimJ, and LldD2) and 3 classical antigens (PPE18, PPE46, and ESAT-6).

To identify features capable of distinguishing between classical and RVMA antigen responses, we applied a Random Forest classification algorithm using the caret package in R and performed the analysis on Cohort 1 and Cohort 2 (see computational analysis methods).

**Figure 4:**
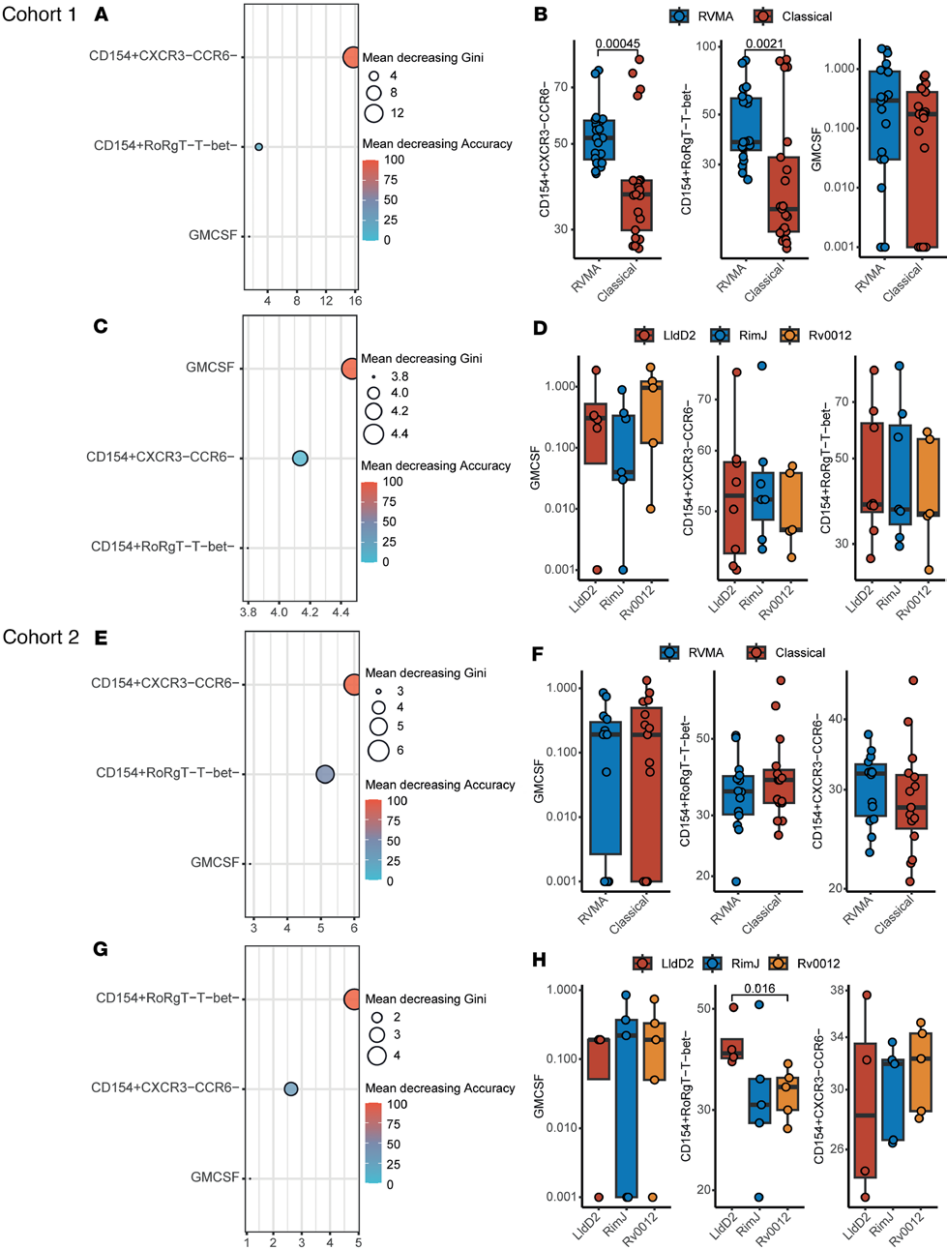
RVMA are enriched for nonclassical Th1 or Th17 responses. (**A**–**H**) Comparison of RVMA and classical antigen-specific antigens marker profile in Cohort 1 (**A**–**D**) and Cohort 2 (**E**–**H**). **A**, **C**, **E**, and **G** show random forest feature selection analysis results. The *y* axis shows the variable names ordered by the Mean Decrease Gini. The *x* axis is displaying the Mean Decrease Gini values, while the color represents the Mean decrease in Accuracy. **B**, **D**, **F**, and **H** show box and whisker plots from the most informative variables identified by the random forest algorithm. The *y* axis shows the variable abundance value transformed in log_10_, while the *x* axis and colors display the antigen groups or antigen identity. Group comparison was performed with the Wilcoxon test, and *P* values were adjusted by FDR.

In Cohort 1, the top marker combination distinguishing RVMA from classical antigens was CD154^+^CCR6^–^CXCR3^–^, followed by CD154^+^T-bet^–^RORγT^–^ and GM-CSF (Figure 4A). The abundance of CD154^+^CCR6^–^CXCR3^–^ and CD154^+^T-bet^–^RORγT^–^ cells was significantly higher for RVMA compared with classical antigens (Figure 4B). Among RVMA antigens, the strongest discriminant was GM-CSF, followed by CD154^+^CCR6^–^CXCR3^–^ and CD154^+^T-bet^–^RORγT^–^ (Figure 4C), though their abundance did not differ significantly across individual antigens (Figure 4D).

In Cohort 2, the key discriminant between RVMA and classical antigens was CD154^+^CCR6^–^CXCR3^–^ followed by CD154^+^T-bet^–^RORγT^–^ and GM-CSF (Figure 4E), with no significant differences in their abundance (Figure 4F). When comparing individual RVMA antigens, CD154^+^T-bet^–^RORγT^–^ emerged as the strongest discriminator, followed by CD154^+^CCR6^–^CXCR3^–^ and GM-CSF (Figure 4G). Notably, CD154^+^T-bet^–^RORγT^–^ cells were significantly more abundant in LldD2 compared with RimJ and Rv0012 responsive cells, while GM-CSF and CD154^+^CCR6^–^CXCR3^–^ levels did not differ among the antigens (Figure 4H). Together, these findings highlight the heterogeneity of CD4 T cell responses induced by RVMA antigens, including nonclassical Th17 and Th1 phenotypes, and underscore the diversity of immune responses in human populations.

### RVMA-responsive CD4 T cells have fewer intermarker interactions than classical antigens

**Figure 5:**
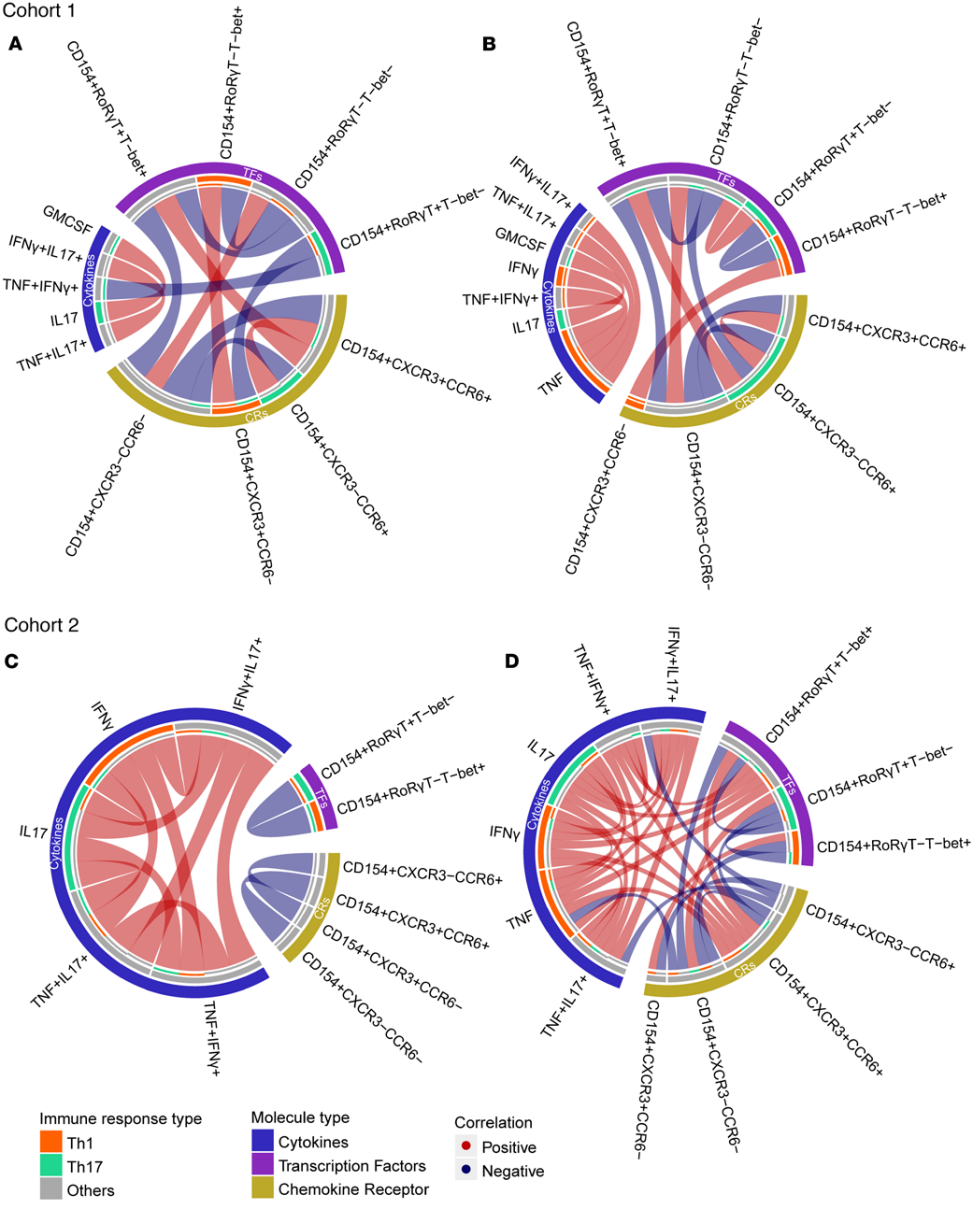
RVMA-responsive CD4 T cells exhibit fewer intermarker interactions. Correlations identified in Cohort 1 (top) and Cohort 2 (bottom), in both RVMA (Rv0012, RimJ, and LldD2) and classical (PPE18, PPE46, and ESAT-6) antigen–stimulated samples. Chord diagrams depicting the correlation identified in RVMA (**A** and **C**) and classical (**B** and **D**) antigens, using Spearman’s correlation analysis. Only correlations with |rho| > 0.4 and FDR < 0.05 were considered. Positive and negative correlations are represented by the red and blue lines, respectively. Cytokines, TFs, and CRs are highlighted in the outer ring as blue, purple, and yellow areas, respectively. Th1 and Th17 variables are highlighted in the inner ring as Orange and Green, respectively.

Finally, we examined associations between cytokines, TFs, and CRs expressed by antigen-responsive CD4 T cells that responded to RVMA (Rv0012, RimJ, and LldD2) or to classical antigens (ESAT-6, PPE18, and PPE46). Analyses were restricted to participants with complete data for all 3 variables. To investigate correlations among cytokines (blue), TFs (purple), and CRs (yellow), and by Th1 (orange) or Th17 (green) subset, we applied Spearman’s correlation separately for each cohort and antigen group (RVMA and classical). Only statistically significant correlations, defined as having an FDR-adjusted *P* < 0.05 and an absolute Spearman’s rho > 0.4, were included. These significant relationships were visualized using chord diagrams (Figure 5, A–D) to highlight the most relevant interactions.

CD4 T cells that responded to RVMA antigens exhibited fewer intermarker network interactions than those responsive to classical antigens. While there were more likely interactions between TFs and CRs, the cytokine interactions were mostly restricted to within the cytokine variable. Consistent with the expected biology, CD154^+^RORγT^–^T-bet^–^ correlated positively with CD154^+^CCR6^–^CXCR3^–^, suggesting *Mtb*-antigen responsive CD4 T cells that are neither Th17 nor Th1, and CD154^+^CXCR3^+^CCR6^–^ correlated positively with CD154^+^RORγT^–^T-bet^+^, indicating a Th1 subset, whereas CD154^+^RORγT^+^T-bet^–^ inversely correlated with TNF-α^+^IFN-γ^+^. Notably, GM-CSF strongly correlated with IL-17, particularly in Cohort 1. These results indicate that *Mtb* antigens influence CD4 T cell differentiation through distinct transcription factor and chemokine receptor expression profiles and highlight a functional link between GM-CSF– and IL-17–producing CD4 T cells.

## Discussion

In 2 cohorts of close HHCs that have controlled *Mtb* infection after recent TB exposure, we discovered that *Mtb* antigens — Rv0010c, Rv0012, RimJ, LldD2, Rv2719c, Rv3221c, and Rv0990c, previously characterized by their unusual (for *Mtb*) presence of T cell epitopes under diversifying evolutionary selection pressure (29, 30) — predominantly induce Th17 immunity, which is distinct from T cell responses induced by classical *Mtb* antigens that are biased toward Th1 differentiation and IFN-γ production. The participants in this study had no signs of TB disease at enrollment and none progressed to TB disease in the 6–12 months (Cohort 1) or 24 months (Cohort 2) of follow-up. On this basis, they can be considered to have controlled *Mtb* infection. We found that CD4 T cells that respond to RVMA express the Th17 lineage–defining transcription factor, RORγT, and secrete IL-17 upon antigen stimulation. While we found that some RVMA-responsive CD4 T cells express CCR6, the chemokine receptor associated with Th17 cells, they express CCR6 in proportions of CD4 T cells similar to those that respond to classical antigens. In contrast, we found that a high proportion of RVMA-responsive CD4 T cells express neither CCR6 nor CXCR3 (the chemokine receptor associated with Th1 cells).

Th17 cells are increasingly appreciated as contributing to *Mtb* control in humans (46–53) and nonhuman primate models of *Mtb* infection or vaccine responses (54–58), but there has been little investigation of the possibility that Th17 cells might preferentially develop in response to distinct *Mtb* antigens. One important study, in which *Mtb* antigens were selected for study in human samples based on their induced expression during infection of multiple strains of mice, reported several antigens that induced IL-17 production measured by ELISA in the absence of detectable IFN-γ (38). Because of different selection criteria, none of those antigens coincide with the antigens we studied here. Together, the findings in that study and the present one emphasize that individual *Mtb* antigens can induce CD4 T cells with distinct effector functions, depending on the specific *Mtb* antigen.

Unlike other infectious pathogens in which the targets of protective immunity undergo antigenic variation to escape immune recognition through diversifying evolutionary selection (17–24), T cell epitopes in the commonly studied classical *Mtb* antigens are highly conserved (29, 30). In this study, we characterized T cells from people protected from progressive/active TB that recognize the rare exceptions: antigens with T cell epitopes that exhibit evidence of diversifying evolutionary selection (variable T cell epitopes) (30), the RVMA. If *Mtb* follows the evolutionary model of other pathogens, then our results suggest that RVMA are antigens whose recognition by host immune responses is especially detrimental to the pathogen, and we found that recognition of RVMA by T cells is common in people who have controlled *Mtb* infection. Further studies will be needed to determine whether recognition of RVMA differs in those who progress to active TB, but those studies will require human cohorts distinct from the ones studied here.

Secreted *Mtb* proteins are widely regarded as promising vaccine candidates due to their strong immunogenicity and ease of heterologous expression and amplification (59, 60). Unlike intracellular proteins, these secreted antigens are actively released by *Mtb* during infection, making them readily accessible to the host immune system. Recent advances in mass spectrometry–based immunopeptidomics have enabled the identification of *Mtb*-derived peptides bound to both MHC-I and MHC-II molecules (61–63). Analyses of peptides presented by *Mtb*-infected human monocyte–derived dendritic cells revealed that most identified antigens originate from secreted proteins, particularly those associated with the Type VII secretion system. Interestingly, some MHC-II–bound peptides failed to elicit measurable IFN-γ or IL-17 responses in *Mtb*-exposed but asymptomatic individuals (63). While immunogenicity is often used to guide vaccine design, several vaccines based solely on highly immunogenic secretory antigens have shown limited success in human trials (64, 65). Notably, neither the RVMA nor the LICA antigens are predicted to be secretory *Mtb* proteins (30, 37).

It has been widely believed that protective immunity to *Mtb* must involve mechanisms (via cytokines or cytotoxicity) that are directed at *Mtb*-infected cells (usually macrophages). However, our results indicate that T cells that recognize RVMA predominantly express IL-17, which is not thought to directly modulate macrophage microbicidal mechanisms. The mechanisms whereby IL-17 contributes to immunity to *Mtb* have not been fully defined. IL-17 is known to induce expression of multiple antimicrobial peptides, and to induce IL-6, G-CSF, and specific chemokines that promote production and migration of neutrophils (66). If IL-17 contributes to immunity to *Mtb* through one of these mechanisms, its role may be predominantly in control of extracellular *Mtb* that have escaped from macrophages. In mouse models of *Mtb* infection, IL-17 has been found to induce expression of the chemokine CXCL13 by nonhematopoietic tissues and to contribute to the formation of T and B cell–enriched cellular aggregates in the lungs that are associated with immune control of *Mtb* (67–70). In another mouse model in which *Mtb* infection is characterized by lung tissue necrosis, IL-17 suppressed HIF-1α and tissue hypoxia and reduced lung inflammation (47). Since evolutionary forces that contribute to pathogen fitness can affect pathogen transmission as well as within-host pathogen survival, it is possible that the effector mechanisms of RVMA-specific T cells predominantly target mechanisms underlying *Mtb* transmission. In addition to inducing antimicrobial peptides and neutrophil-directed chemokines, evidence is emerging that IL-17 contributes to tissue homeostasis and repair (reviewed in ref. 66). Since inflammatory lung tissue destruction and cavitation contribute to *Mtb* transmission (71), our results suggest that T cells that target RVMA and produce IL-17 may have their predominant effects by countering lung tissue destruction. Since pathogen transmission is an important determinant of evolutionary success, antigenic variation to enable *Mtb* to escape recognition by T cells that produce IL-17 and prevent lung tissue damage may account for the sequence diversity of RVMA.

The role of GM-CSF in human *Mtb* infection outcomes remains poorly understood. In mice, IFN-γ–independent *Mtb* control requires CD4 T cell–derived GM-CSF and activation of HIF-1α (72). *Mtb* lysate stimulation induced GM-CSF production by CD4 T cells from individuals with active TB (45), demonstrating its involvement in *Mtb*-specific T cell responses. Notably, GM-CSF responses to ESAT-6/CFP-10 and Rv1733c were diminished 1–2 years before TB progression in IGRA^+^ HIV-infected individuals compared with nonprogressors (73), suggesting that GM-CSF responses may serve as early correlates of protection. However, additional longitudinal studies are necessary to define the role of GM-CSF–producing T cells in *Mtb* infection outcome. We checked for bifunctional (IL-17^+^GM-CSF^+^) CD4 T cells but found no consistent coproduction of both cytokines in our cohorts, despite the observation that the donors with detectable IL-17 responses also likely had detectable GM-CSF. As a result, we cannot make firm conclusions on the link between IL-17 and GM-CSF.

Effective *Mtb* control requires diverse T cell phenotypes beyond classical Th1 and emerging Th17 subsets. In HHCs who remain TST^–^ and IGRA^–^ without TB symptoms, antigen-specific T-bet^–^RORγT^–^ CD4 T cells were enriched, implicating a nonclassical Th1/Th17 phenotype in *Mtb* containment (53). Moreover, regulatory T cells (CD4^+^FoxP3^+^) are increased in human TB granulomas from individuals with active TB (74) and enriched among *Mtb* antigen–activated CD4 T cells in IGRA^–^ compared with IGRA^+^ individuals (53). Although Tregs were not evaluated in the present study, future work should explore the identity of CCR6^–^CXCR3^–^ and T-bet^–^RORγT^–^ RVMA-specific CD4 T cells.

Further work will be required to define the mechanisms whereby distinct *Mtb* antigens drive divergent T cell differentiation programs. Potential mechanisms include differences in antigen-presenting cell activation and cytokine milieu, variation in T cell receptor affinity, and whether T cells are primed directly by infected dendritic cells or indirectly through antigen transfer to uninfected cells. Given the complexity and heterogeneity of immune responses to *Mtb*, effective control likely depends on inducing specific properties of antigen-specific CD4 T cells (75) that future vaccines should aim to recapitulate. Converging evidence from human studies (43, 51, 53), animal infection models (57, 58), and vaccination experiments (54–56, 76, 77) indicates an important role for Th17 responses in *Mtb* control. Our study advances TB vaccine research efforts by identifying a class of *Mtb* antigens that preferentially elicit Th17 responses in *Mtb*-exposed individuals.

The studies reported here have several limitations. First, although they involved participants in 2 distinct cohorts in East Africa, they may not be generalizable to other populations. Second, they do not directly reveal whether RVMA-specific human T cells uniquely contribute to protective immunity to *Mtb*; further studies in cohorts that compare responses to RVMA in those with active versus controlled *Mtb* infection are needed. Third, they do not establish mechanisms whereby RVMA-responsive T cells that produce IL-17 contribute to immunity to *Mtb*.

In summary, we provide unique evidence that *Mtb* antigens with the rare property of undergoing diversifying selection drive the development of CD4 T cells with functional properties distinct from T cells that recognize “classical” conserved *Mtb* antigens. Our results demonstrate that T cells that recognize RVMA exhibit a Th17 phenotype, which has been reported to provide beneficial contributions to human immunity to TB. RVMA may be especially valuable antigens in vaccines designed to amplify Th17 responses that resulted from initial infection. Thus, including RVMA in vaccines designed to prevent progression to Mtb disease may broaden the range of T cell effector functions achieved by vaccination and further reduce the risk of progression to active TB.

## Methods

### Sex as a biological variable

PBMC were isolated from blood samples collected from both male and female participants aged 13 years and older for ELISA and flow cytometry analyses. When data were stratified by sex, no significant differences were observed between male and female participants.

### Study participants and sample collection

#### Cohort 1.

HHCs of newly diagnosed active pulmonary TB cases were referred to the Kenya Medical Research Institute (KEMRI) Clinical Research Center in Kisumu, Kenya, and their demographic and medical history data were collected. Active pulmonary TB (index) cases with drug-susceptible TB were symptomatic individuals with acid-fast bacilli (AFB) sputum smear positive or a positive GeneXpert MTB/RIF (Cepheid) result and a positive culture for *Mtb* growth identified at community health clinics in Kisumu, Western Kenya. HHCs were persons who shared the same home residence as the index case for ≥ 5 nights during the 30 days prior to the date of TB diagnosis of the index case and were enrolled no more than 3 months (mean: 18 days; range: 1–77 days) after the index case began TB treatment. All participants provided written informed consent to join the study and were recruited from 2 community-based health clinics located in Kisumu City and Kombewa, Kisumu County. All enrolled individuals met the following inclusion criteria: ≥ 13 years of age at the time of enrollment, positive QuantiFERON TB Gold in Tube (QFT) result, seronegative for HIV antibodies, no previous history of diagnosis or treatment for active TB disease or LTBI, normal chest X-ray, and not pregnant. All participants were presumed to be Bacillus Calmette-Guérin (BCG) vaccinated due to the Kenyan policy of BCG vaccination at birth and high BCG coverage rates throughout Kenya.

#### Cohort 2.

Enrollment criteria and procedures for this cohort were the same as for Cohort 1, with the difference that potential participants (index cases and HHCs) were identified through public health surveillance at community health facilities in Addis Ababa, Ethiopia.

At both study locations, blood samples were collected from participants in sodium heparin or lithium heparin Vacutainer CPT Mononuclear Cell Preparation Tubes (BD Biosciences or Greiner Bio-One). PBMC were isolated by density centrifugation, rested in complete media (RPMI 1640 containing L-glutamine supplemented with 10% heat-inactivated fetal bovine serum [FBS], 1% penicillin-streptomycin [PenStrep], 1% Hepes) before counting, and stored in liquid nitrogen (LN_2_) until use. PBMC isolation was initiated < 2 hours after the blood was drawn. Isolated PBMC were cryopreserved in 90% heat-inactivated fetal calf serum/10% DMSO and kept in LN_2_ until they were thawed for study at the UCSF laboratory.

### Mtb antigens

Sixty distinct *Mtb* antigens were synthesized as peptide pools of 18 amino acids overlapping by 11, as previously described (37). These antigens are derived from proteins in different bacterial functional categories, including cell wall and cell processes; intermediary metabolism and respiration; virulence, detoxification, and adaptation; information pathways; lipid metabolism; and conserved hypotheticals. Additionally, the antigens are derived from different fractions of the bacteria, including membrane, secreted, cytoplasm, cell wall, and predicted membrane/secreted proteins. Several of the antigens were initially identified by their exhibiting evidence of antigenic variation and diversifying evolutionary selection pressure. For those antigens, the overlapping peptide pools included all of the known sequence variants identified in ref. 30.

### Whole blood response spectrum assay (RSA)

Heparinized whole blood was processed within 2 hours of collection as previously described (37). Blood was diluted at 1:4 with media consisting of RPMI 1640 medium supplemented with 2 mM L-glutamine, 100 U/mL penicillin, and 100 mg/mL streptomycin. Diluted blood (100 μL) was added to each well of a sterile 96-well round-bottom, tissue culture–treated plate (Corning) containing 100 μL of RSA medium alone (negative control), RSA medium with individual *Mtb* antigen prepared as peptide pools at 1 μg/mL final concentration, or RSA medium with PHA (positive control) to make a final volume of 200 μL per well. Plates were incubated in a 37°C incubator with 5% CO_2_ for 7 days. On day 7, plates were centrifuged at 900*g* for 5 minutes, and 150 μL of supernatant was removed from each well and transferred to a V-bottom 96-well plate (Corning). Plasma supernatants were stored at –80°C until use for ELISAs. The assays were performed in laboratories at KEMRI, in Kisumu, Kenya.

### IFN-γ ELISA

IFN-γ in supernatants from the whole blood RSA was determined by ELISA according to the manufacturer’s instructions (Human IFN-γ Uncoated ELISA kit; KHC4021, Invitrogen). In total, 50 μL of supernatant was diluted with 50 μL of assay diluent for use in the IFN-γ ELISA. ELISA plates were read at 450 nm (Synergy H1 Microplate reader, Biotek), and data collected using Gen5 software (Biotek).

### PBMC antigen stimulation and ICS

Cryopreserved PBMC were thawed in a 37°C water bath until there were ice balls in the cryotubes; they were then quickly transferred into prewarmed R10 media (RPMI 1640 containing L-glutamine supplemented with 10% FBS, 1% PenStrep and 1% Hepes). Cells were centrifuged at 900*g* for 5 minutes at room temperature, the supernatant was discarded, and then cells were resuspended before adding 5 mL of warm R10 media and transferred to a 6-well culture plate overnight at 37°C/5% CO_2_.

Rested cells were transferred to 15 mL tubes and live cells were counted using Trypan blue exclusion. In total, 1 × 10^6^ live cells in 200 μL R10 were transferred to each well of a 96-well round bottom culture plate and stimulated with peptide pools (2 μg/mL) representing single *Mtb* antigens or SEB as a positive control (1 μg/mL). Unstimulated samples were included for each participant as negative controls. Anti-CD28 (348040, 1 μg/mL) and anti-CD49d (340976, 1 μg/mL) costimulatory antibodies (both BD Biosciences) were added to each well and the cells were incubated for 2 hours before adding GolgiStop (catalog 554724) and GolgiPlug (catalog 555029) (both from BD Biosciences). The cells were incubated for an additional 18 hours.

After 20 hours of total stimulation, cells were centrifuged at 900*g* for 5 minutes at room temperature and stained with Zombie Aqua Fixable viability kit (catalog 423101, BioLegend, 1:1,000 diluted in PBS, 100 μL) for 20 minutes at room temperature in the dark to enable exclusion of dead cells. After incubation, 100 μL of PBS was added to each well and centrifuged at 900*g* for 5 minutes at room temperature. Cells were washed one more time with 200 μL PBS, and supernatants were discarded. Next, the cells were resuspended in 50 μL of surface antibody cocktail diluted in Brilliant Stain buffer (563794, BD Biosciences) for 20 minutes in the dark. The surface antibody cocktail consisted of αCD3 Brilliant Violet 421 (clone UCHT1, BioLegend) or PE-CF594 (clone UCHT1, BD Biosciences), αCD4 Brilliant Violet 605 (clone SK3, BioLegend), αCD8 Brilliant Violet 650 (clone RPA-T8, BioLegend) or Brilliant Violet 570 (clone RPA-T8, BioLegend), αCD45RA Pacific Blue (clone HI100, BioLegend), αCCR7 Brilliant Violet 785 (clone G043H7, BioLegend), αCD95 PerCP/Cyanine5.5 (clone DX2, BioLegend) and αCD27 FITC (clone O323, BioLegend). An additional 150 μL of PBS was added into each well, and cells were centrifuged at 900*g* for 5 minutes at room temperature; the supernatant was discarded, and cells were washed again with 200 μL PBS. Cells were then permeabilized and fixed using BD Cytofix/Cytoperm kit (554714, BD Biosciences) according to the manufacturer’s instructions for 20 minutes at 4°C protected from light. Cells were washed twice (900*g* for 5 minutes each) with 1X BD Perm/Wash solution. Cells were resuspended in 50 μL intracellular antibody cocktail diluted in 1X BD Perm/Wash for 20 minutes at room temperature. The cytokine staining cocktail consisted of αTNF Alexa Fluor 700 clone MAb11 (BD Biosciences), αIFN-γ APC/Cyanine7 clone 4S.B3 or PE/Cyanine7 clone 4S.B3 (both BioLegend), αGM-CSF APC (clone MP1-22E9, BioLegend), and αIL-17 PE (BL168, BioLegend). After intracellular staining, cells were washed twice with 1X BD Perm/Wash solution. Cells were then fixed in 2% PFA and acquired with an LSR-II cytometer (BD Biosciences).

### T cell AIM assay

Cryopreserved PBMC were thawed and rested overnight, and 1 × 10^6^ live cells in 200 μL R10 were transferred to each well of a 96-well round-bottom culture plate. Costimulatory antibodies anti-CD28 (1 μg/mL) and anti-CD49d (1 μg/mL) together with anti-CD40 (130-108-041, 1 μg/mL, Miltenyi Biotec) were added into each well and rested for 15 minutes in 37°C/5%CO_2_ incubator. Next, peptide pools representing single *Mtb* antigens at a final concentration of 2 μg/mL and SEB positive control (1 μg/mL) were used to stimulate the cells for a total of 20 hours. Wells with no stimulus were included as negative controls for each participant in each assay. At the end of stimulation, cells were centrifuged at 900*g* for 5 minutes at room temperature and surface stained with Live/Dead Fixable Blue Dead Cell stain kit (L23105, Invitrogen) for 20 minutes at room temperature in the dark. Cells were washed twice (900*g* for 5 minutes at room temperature) and then 50 μL of surface antibody cocktail diluted in Brilliant Stain buffer (563794, BD Biosciences) added and incubated 20 minutes at room temperature in the dark. The surface antibody cocktail consisted of αCD3 Brilliant Violet 510 (clone UCHT1, BioLegend), αCD4 BUV496 (clone SK3, BD Biosciences), αCD8 Brilliant Violet 570 (clone RPA-T8, BioLegend), αCD154 PE-CF594 (clone TRAP1, BD Biosciences), αCCR6 FITC (clone G034E3, BioLegend) and αCXCR3 Brilliant Violet 650 (clone G025H7, BioLegend). After adding 150 μL of PBS to each well, the cells centrifuged at 900*g* for 5 minutes at room temperature, supernatant was discarded, and cells were washed again with 200 μL PBS. Cells were then permeabilized and fixed using eBioscience FOXP3/Transcription Factor staining kit (00-5523-00, Invitrogen) according to the manufacturer’s instructions for 20 minutes at 4°C protected from light. Cells were washed twice with 1X eBioscience Perm diluent and then resuspended in 50 μL of intracellular antibody mix of αRORγT Alexa Fluor 647 (clone Q21-559, BD Biosciences) and αT-bet PE-Dazzle 594 (clone 4B10) or PE (clone 4B10) (both BioLegend) diluted in eBiosciences perm diluent. After TF staining, cells were washed twice with 1X eBioscience Perm diluent. Cells were fixed in 2% PFA and acquired using a 5-Laser Cytek Aurora Spectral Flow cytometer (Cytek Biosciences).

### Computational analysis

Data from each cohort were compiled by variable type (cytokines, TFs, and CRs) and merged to generate a complete dataset encompassing both RVMA and classical antigens. Antigens were grouped as RVMA (Rv0012, RimJ, and LldD2) or classical (PPE18, PPE46, and ESAT-6). The final merged dataset included 41 samples for Cohort 1 and 29 samples for Cohort 2.

#### Feature selection.

To identify immune features distinguishing RVMA from classical antigens, we used a Random Forest classification approach implemented with the *caret* package in R (78, 79). Analyses were performed separately for each cohort using 2 strategies: (a) comparison between the 2 antigen groups (RVMA versus classical) and (b) discrimination among individual RVMA antigens. The Random Forest model was trained using the following parameters: ntree = 1000, nPerm = 10, and nodesize = 1. Cross-validation was performed using the trainControl function with method = “repeatedcv”, repeats = 5, and number = 20. Model performance was evaluated through confusion matrices comparing predicted and actual classifications. Informative features were ranked according to their Mean Decrease in Gini index. Feature abundance values were log_10_ transformed and visualized using box plots. Statistical comparisons between antigen groups were conducted using the Wilcoxon test (FDR-adjusted *P* values) and the Kruskal-Wallis test.

#### Correlation analysis.

To assess the relationships among cytokines, TFs, and CRs, we performed Spearman’s correlation analysis separately for each cohort and antigen group (RVMA and classical), generating 4 distinct correlation matrices: Cohort 1 RVMA, Cohort 1 Classical, Cohort 2 RVMA, and Cohort 2 Classical. Statistically significant correlations were defined by an FDR-adjusted *P* < 0.05 and an absolute Spearman’s rho > 0.4. Significant associations were then visualized as chord diagrams using the circlize package in R, allowing for the identification and comparison of key immune network interactions across antigen classes and cohorts (80).

### Statistics

IFN-γ ELISA data were analyzed using SoftMax Pro v6.3 software (Molecular Devices). IFN-γ release for each antigen was determined by subtracting the mean background IFN-γ concentration in 6 negative control wells lacking antigens for each participant sample. A maximum concentration of quantifiable IFN-γ was set at 1,000 pg/mL, corresponding to the concentration of the highest standard of recombinant human IFN-γ (Human IFN-γ Gamma Uncoated ELISA kit, Invitrogen). IFN-γ concentrations below the level of detection by the ELISA standard curve were set to 0 pg/mL. Spectral flow fcs files were initially analyzed using SpectroFlo software v3.0 (Cytek Biosciences) for unmixing and autofluorescence correction. Identification of distinct T cell populations from unmixed fcs files (spectral flow data) and compensated fcs files (LSR-II data) was performed using Flowjo v10 (Flowjo LLC). Antigen-specific cytokine-producing CD4 T cell magnitudes are reported after subtraction of the values from unstimulated samples for each participant; results greater than those of unstimulated samples were considered positive, with the limit of detection set at 0.001% of total CD4 T cells. All statistical analyses were performed using GraphPad Prism version 9.0 or 10 (GraphPad Software Inc). Comparisons of 2 groups were done by a paired or unpaired 2-tailed Student’s *t* test (Wilcoxon’s test or Mann-Whitney *U* test, respectively), and *P* < 0.05 was considered statistically significant. Comparison of 3 or more groups was done using ANOVA, followed by multiple comparisons as applicable and reported in each corresponding figure. For the calculation of the response magnitudes, samples that yielded undetectable results were entered as 0.001% of CD4 T cells.

### Study approval

All participants gave written informed consent for the study, which was approved by the respective institutional Scientific and Ethical Review Committees. Cohort 1: KEMRI/CDC Scientific and Ethics Review Unit; Cohort 2: AHRI/ALERT Ethics Review Committee; and the Ethiopian National Research Ethics Review Committee and the Institutional Review Board at Emory University approved studies for both cohorts.

### Data availability

All deidentified data are available from the corresponding authors upon request. Values for all data points in graphs are reported in the Supporting Data Values file.

## Author contributions

PO designed research studies, conducted experiments, acquired data, analyzed data, created figures, wrote the initial draft, and reviewed and edited the manuscript. LW, GO, SGO, and KB contributed project administration, participant recruitment, acquiring data, and reviewing and editing the manuscript. AT and JH conducted experiments, acquired data, and reviewed and edited the manuscript. DC and LS contributed project administration and reviewing and editing the manuscript. CSLA, NRG, JR, CLD, and JDA contributed study conceptualization, project administration, funding acquisition, and reviewing and editing the manuscript. SCA analyzed data and reviewed and editing the manuscript. AQ, MAP, EF, BBA analyzed data, created figures, and reviewed and edited the manuscript. HMB contributed study conceptualization, project administration, funding acquisition, and reviewing and editing the manuscript. JDE contributed study conceptualization, designing research studies, project administration, analyzing data, reviewing and editing the manuscript, funding acquisition, and supervision. TBRU Astra Study Group contributed to participant recruitment and clinical data analysis.

## Funding support

This work is the result of NIH funding, in whole or in part, and is subject to the NIH Public Access Policy. Through acceptance of this federal funding, the NIH has been given a right to make the work publicly available in PubMed Central.

- NIH/NIAID Grant U19AI111211 Tuberculosis Research Unit (TBRU) (MPIs: HMB/JDE).
- NIH/NIAID grant R01 AI173002 (PI: JDE).
- UCSF-CFAR (P30 AI027763; to PO).
- NIAID-UC-TRAC P30AI168440 (to PO).
- NIH/NIAID R25AI147375 (to PO).
- Fellowship from the Helen Hay Whitney Foundation (to PO).
- Emory/Georgia TRAC 1P30AI168386 (MPIs: NRG/JR.)
- K24 grant K24AI114444 (PI: NRG).

## Supplementary Material

Supplemental data

ICMJE disclosure forms

Supporting data values

## Figures and Tables

**Table 1:**
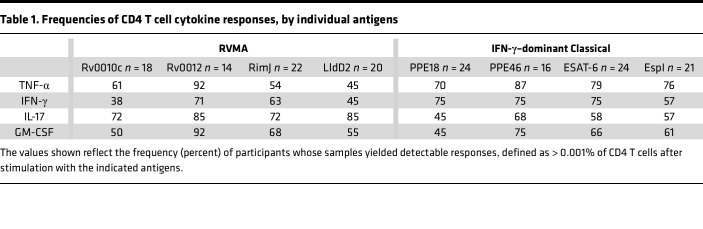
Frequencies of CD4 T cell cytokine responses, by individual antigens

**Table 2:**
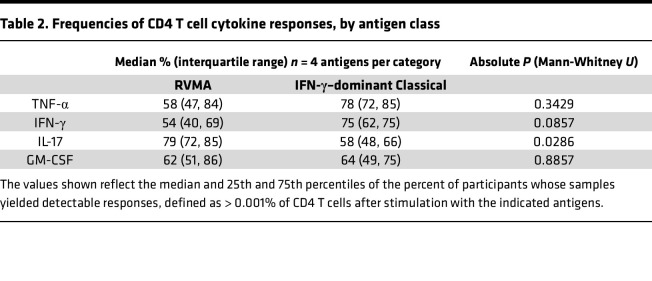
Frequencies of CD4 T cell cytokine responses, by antigen class

**Table 3:**
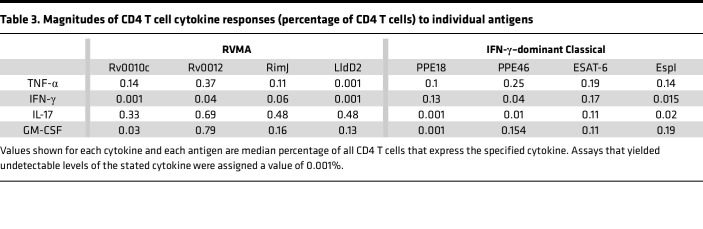
Magnitudes of CD4 T cell cytokine responses (percentage of CD4 T cells) to individual antigens

**Table 4:**
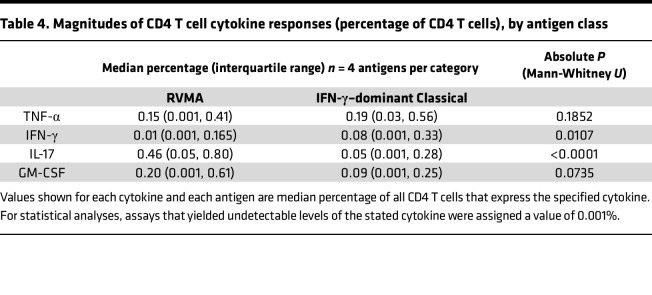
Magnitudes of CD4 T cell cytokine responses (percentage of CD4 T cells), by antigen class

## Version 1 01/27/2026

In-Press Preview

## Version 2 03/23/2026

Electronic publication
