# Supplementary material for: Rare variable M. tuberculosis antigens induce predominant Th17 responses in human infection
Source: JCI Insight. 2026 Jan 27;11(6):e202134. doi: 10.1172/jci.insight.202134 (PMC13043089; doi:10.1172/jci.insight.202134)
Supplement: Supplemental data [file jciinsight-11-202134-s317.pdf]

Rare Variable *M. tuberculosis* Antigens induce predominant Th17 responses in human infection

Paul Ogongo[1,2*], Liya Wassie[3], Anthony Tran[1], Devin Columbus[1#], Julia Huffaker[1], Lisa Sharling[4], Gregory Ouma[5], Samuel Gurrion Ouma[5], Kidist Bobosha[3], Cecilia S. Lindestam Arlehamn[6,7], Neel R. Gandhi[4,8,9], Sara C. Auld[4,9,12], Jyothi Rengarajan[8,10], Cheryl L. Day[10,11], Artur Quieroz[13,14], Mariana Araújo-Perreira[13,14], Eduardo Fukutani[13,14], Bruno B. Andrade[13,14], John D. Altman[10], Henry M. Blumberg[4,8,9], Joel D. Ernst[1*] and the TBRU ASTRa Study Group

[1]Division of Experimental Medicine, University of California, San Francisco, CA, USA

[2]Department of Tropical and Infectious Diseases, Kenya Institute of Primate Research, Nairobi, Kenya

[3]Mycobacterial Disease Research Directorate, Armauer Hansen Research Institute, Addis Ababa, Ethiopia

[4]Department of Epidemiology, Emory University Rollins School of Public Health, Atlanta, GA, USA

[5]Center for Global Health Research, Kenya Medical Research Institute, Kisumu, Kenya

[6]Center for Vaccine Innovation, La Jolla Institute for Immunology, La Jolla, CA, USA

[7]Department of Infectious Disease and Immunology, Center for Vaccine Research, Statens Serum Institut, Copenhagen, Denmark.

[8]Department of Medicine, Division of Infectious Diseases, Emory University School of Medicine, Atlanta, GA, USA

[9]Department of Global Health, Emory University Rollins School of Public Health, Atlanta, GA, USA,

[10]Emory Vaccine Center, Emory University, Atlanta, GA, USA

[11]Department of Microbiology and Immunology, Emory University School of Medicine, Atlanta, GA, USA

[12]Department of Medicine, Division of Pulmonary and Critical Care Medicine, Emory University School of Medicine, Atlanta, GA, USA

[13]Multinational Organization Network Sponsoring Translational and Epidemiological Research (MONSTER) Initiative, Salvador, Brazil

[14]Laboratório de Pesquisa Clínica e Translacional, Instituto Gonçalo Moniz, Fundação Oswaldo Cruz, Salvador, Brazil

#Present address: Vitamin Angels Alliance, Goleta, CA, USA
*Joint corresponding authors:
Paul Ogongo:
University of California, San Francisco,
2540 23rd Street, Pride Hall, Room 3724
San Francisco, CA 94110
tel: 415-476-1227
paul.ogongo@ucsf.edu

Joel D. Ernst:
University of California, San Francisco,
2540 23rd Street, Pride Hall, Room 3724
San Francisco, CA 94110
tel: 415-476-1227
joel.ernst@ucsf.edu

Tuberculosis Research Unit – Role of Antigen Specific T Cell Responses in the Control of TB (TBRU- ASTRa) Consortium Study members:

Name	Affiliation
Rafi Ahmed	Emory Vaccine Center, Emory University, Atlanta, GA, USA
Lance Waller	Department of Biostatistics and Bioinformatics, Rollins School of Public Health, Emory University, Atlanta, GA USA
Lisa Elon	Department of Biostatistics and Bioinformatics, Rollins School of Public Health, Emory University, Atlanta, GA USA
Andrea Knezevic	Department of Biostatistics and Bioinformatics, Rollins School of Public Health, Emory University, Atlanta, GA USA
Shirin Jabbarzadeh	Department of Biostatistics and Bioinformatics, Rollins School of Public Health, Emory University, Atlanta, GA USA
Azhar Nizam	Department of Biostatistics and Bioinformatics, Rollins School of Public Health, Emory University, Atlanta, GA USA
Hao Wu	Department of Biostatistics, Rollins School of Public Health, Emory University, Atlanta, GA 30322
Seegar Swanson	Department of Biostatistics, Rollins School of Public Health, Emory University, Atlanta, GA 30322
Yunyun Chen	Department of Biostatistics, Rollins School of Public Health, Emory University, Atlanta, GA 30322
Wendy Whatney	Emory Vaccine Center, Emory University, Atlanta, GA, USA
Melanie Quezada	Emory Vaccine Center, Emory University, Atlanta, GA, USA
Loren Sasser	Emory Vaccine Center, Emory University, Atlanta, GA, USA
Ranjna Madan Lala	Emory Vaccine Center, Emory University, Atlanta, GA, USA
Tawania Fergus	Department of Medicine, New York University School of Medicine, New York, NY, USA. Present address: Division of Rheumatology, Albert Einstein College of Medicine, Bronx, NY
Toidi Adekambi	Emory Vaccine Center, Emory University School of Medicine
Deepak Kaushal	Texas Biomedical Research Institute, San Antonio, TX, USA
Nadia Golden	Tulane National Primate Research Center, Tulane University School of Medicine, Covington, LA, USA
Taylor Foreman	Tulane National Primate Research Center, Tulane University School of Medicine, Covington, LA, USA
Allison Bucsan	Tulane National Primate Research Center, Tulane University School of Medicine, Covington, LA, USA. Present address: Vaccine Research Center, National Institute of Allergy and Infectious Diseases
Chris Ibegbu	Emory Vaccine Center, Emory University, Atlanta, GA, USA
Susanna Contraras Alcantra	Emory Vaccine Center, Emory University, Atlanta, GA, USA
Alessandro Sette	Center for Vaccine Innovation, La Jolla Institute for Immunology, La Jolla, CA, USA

Salim Allana	Department of Epidemiology, Emory University Rollins School of Public Health, Atlanta, GA, USA
Angela Campbell	Department of Epidemiology, Emory University Rollins School of Public Health, Atlanta, GA, USA
Sarita Shah	Department of Epidemiology, Emory University Rollins School of Public Health, Atlanta, GA, USA
Susan Ray	Division of Infectious Diseases, Department of Medicine, Emory University School of Medicine, Atlanta, Georgia, USA.
James Brust	Division of Infectious Diseases, Department of Medicine, Albert Einstein College of Medicine and Montefiore Medical Center, Bronx, New York, USA.
Jeffrey M. Collins	Division of Infectious Diseases, Department of Medicine, Emory University School of Medicine, Atlanta, Georgia, USA.
Meghan Franczek	Department of Epidemiology, Emory University Rollins School of Public Health, Atlanta, GA, USA
Jenna Daniel	Department of Epidemiology, Emory University Rollins School of Public Health, Atlanta, GA, USA
Alison GC Smith	Department of Epidemiology, Emory University Rollins School of Public Health, Atlanta, GA, USA
Anirudh Rao	Department of Epidemiology, Rollins School of Public Health, Emory University
Rebecca Goldstein	Department of Epidemiology, Rollins School of Public Health, Emory University
Madeleine Kabongo	Department of Epidemiology, Emory University Rollins School of Public Health, Atlanta, GA, USA
Alawode Oladele	DeKalb County Board of Health, Atlanta, GA, USA
Janet Agaya	Center for Global Health Research, Kenya Medical Research Institute, Kisumu, Kenya
Jeremiah Khayumbi	Center for Global Health Research, Kenya Medical Research Institute, Kisumu, Kenya
Joan Tonui	Center for Global Health Research, Kenya Medical Research Institute, Kisumu, Kenya
Benson Muchiri	Center for Global Health Research, Kenya Medical Research Institute, Kisumu, Kenya
Joshua Ongalo	Center for Global Health Research, Kenya Medical Research Institute, Kisumu, Kenya
Dickson Gethi	Center for Global Health Research, Kenya Medical Research Institute, Kisumu, Kenya
Felix Hayara Odhiambo	Center for Global Health Research, Kenya Medical Research Institute, Kisumu, Kenya
Dorine Awilly	Center for Global Health Research, Kenya Medical Research Institute, Kisumu, Kenya
Albert Ochieng Okumu	Center for Global Health Research, Kenya Medical Research Institute, Kisumu, Kenya
Abraham Aseffa	Mycobacterial Disease Research Directorate, Armauer Hansen Research Institute, Addis Ababa, Ethiopia

Medina Hamza	Mycobacterial Disease Research Directorate, Armauer Hansen Research Institute, Addis Ababa, Ethiopia
Yonas Abebe	Mycobacterial Disease Research Directorate, Armauer Hansen Research Institute, Addis Ababa, Ethiopia
Fisseha Mulate	Mycobacterial Disease Research Directorate, Armauer Hansen Research Institute, Addis Ababa, Ethiopia
Mekdelawit Wondiyfraw	Mycobacterial Disease Research Directorate, Armauer Hansen Research Institute, Addis Ababa, Ethiopia
Firaol Degaga	Mycobacterial Disease Research Directorate, Armauer Hansen Research Institute, Addis Ababa, Ethiopia
Daniel Getachew	Mycobacterial Disease Research Directorate, Armauer Hansen Research Institute, Addis Ababa, Ethiopia
Dawit Tayachew Bere	Mycobacterial Disease Research Directorate, Armauer Hansen Research Institute, Addis Ababa, Ethiopia
Meaza Zewdu	Mycobacterial Disease Research Directorate, Armauer Hansen Research Institute, Addis Ababa, Ethiopia
Daniel Mussa	Mycobacterial Disease Research Directorate, Armauer Hansen Research Institute, Addis Ababa, Ethiopia
Bezalam Tesfaye	Mycobacterial Disease Research Directorate, Armauer Hansen Research Institute, Addis Ababa, Ethiopia
Selam Jemberu	Mycobacterial Disease Research Directorate, Armauer Hansen Research Institute, Addis Ababa, Ethiopia
Azeb Tarekegn	Mycobacterial Disease Research Directorate, Armauer Hansen Research Institute, Addis Ababa, Ethiopia
Gebeyehu Assefa	Mycobacterial Disease Research Directorate, Armauer Hansen Research Institute, Addis Ababa, Ethiopia
Gutema Jebessa	Mycobacterial Disease Research Directorate, Armauer Hansen Research Institute, Addis Ababa, Ethiopia
Zewdu Solomon	Mycobacterial Disease Research Directorate, Armauer Hansen Research Institute, Addis Ababa, Ethiopia
Sebsibe Neway	Mycobacterial Disease Research Directorate, Armauer Hansen Research Institute, Addis Ababa, Ethiopia
Jemal Hussein	Mycobacterial Disease Research Directorate, Armauer Hansen Research Institute, Addis Ababa, Ethiopia
Tsegaye Hailu	Mycobacterial Disease Research Directorate, Armauer Hansen Research Institute, Addis Ababa, Ethiopia
Alemayehu Geletu	Mycobacterial Disease Research Directorate, Armauer Hansen Research Institute, Addis Ababa, Ethiopia
Edom Girma	Mycobacterial Disease Research Directorate, Armauer Hansen Research Institute, Addis Ababa, Ethiopia
Million Legesse	Mycobacterial Disease Research Directorate, Armauer Hansen Research Institute, Addis Ababa, Ethiopia
Mitin Wendaferew	Mycobacterial Disease Research Directorate, Armauer Hansen Research Institute, Addis Ababa, Ethiopia
Hirut Solomon	Mycobacterial Disease Research Directorate, Armauer Hansen Research Institute, Addis Ababa, Ethiopia

Zenebech Assefa	Mycobacterial Disease Research Directorate, Armauer Hansen Research Institute, Addis Ababa, Ethiopia
Mahlet Mekuria	Mycobacterial Disease Research Directorate, Armauer Hansen Research Institute, Addis Ababa, Ethiopia
Misker Kedir	Mycobacterial Disease Research Directorate, Armauer Hansen Research Institute, Addis Ababa, Ethiopia
Eleni Zeleke	Mycobacterial Disease Research Directorate, Armauer Hansen Research Institute, Addis Ababa, Ethiopia
Rediet Zerihun	Mycobacterial Disease Research Directorate, Armauer Hansen Research Institute, Addis Ababa, Ethiopia
Selam Dechasa	Mycobacterial Disease Research Directorate, Armauer Hansen Research Institute, Addis Ababa, Ethiopia
Emebet Haile	Mycobacterial Disease Research Directorate, Armauer Hansen Research Institute, Addis Ababa, Ethiopia
Nahom Getachew	Mycobacterial Disease Research Directorate, Armauer Hansen Research Institute, Addis Ababa, Ethiopia
Firaol Wagari	Mycobacterial Disease Research Directorate, Armauer Hansen Research Institute, Addis Ababa, Ethiopia
Ruth Mekonnen	Mycobacterial Disease Research Directorate, Armauer Hansen Research Institute, Addis Ababa, Ethiopia
Samuel Bayu	Mycobacterial Disease Research Directorate, Armauer Hansen Research Institute, Addis Ababa, Ethiopia
Melat Gebre-Medhin	Mycobacterial Disease Research Directorate, Armauer Hansen Research Institute, Addis Ababa, Ethiopia
Alemayehu Kifle	Mycobacterial Disease Research Directorate, Armauer Hansen Research Institute, Addis Ababa, Ethiopia

Supplementary materials

Supplementary figure 1:

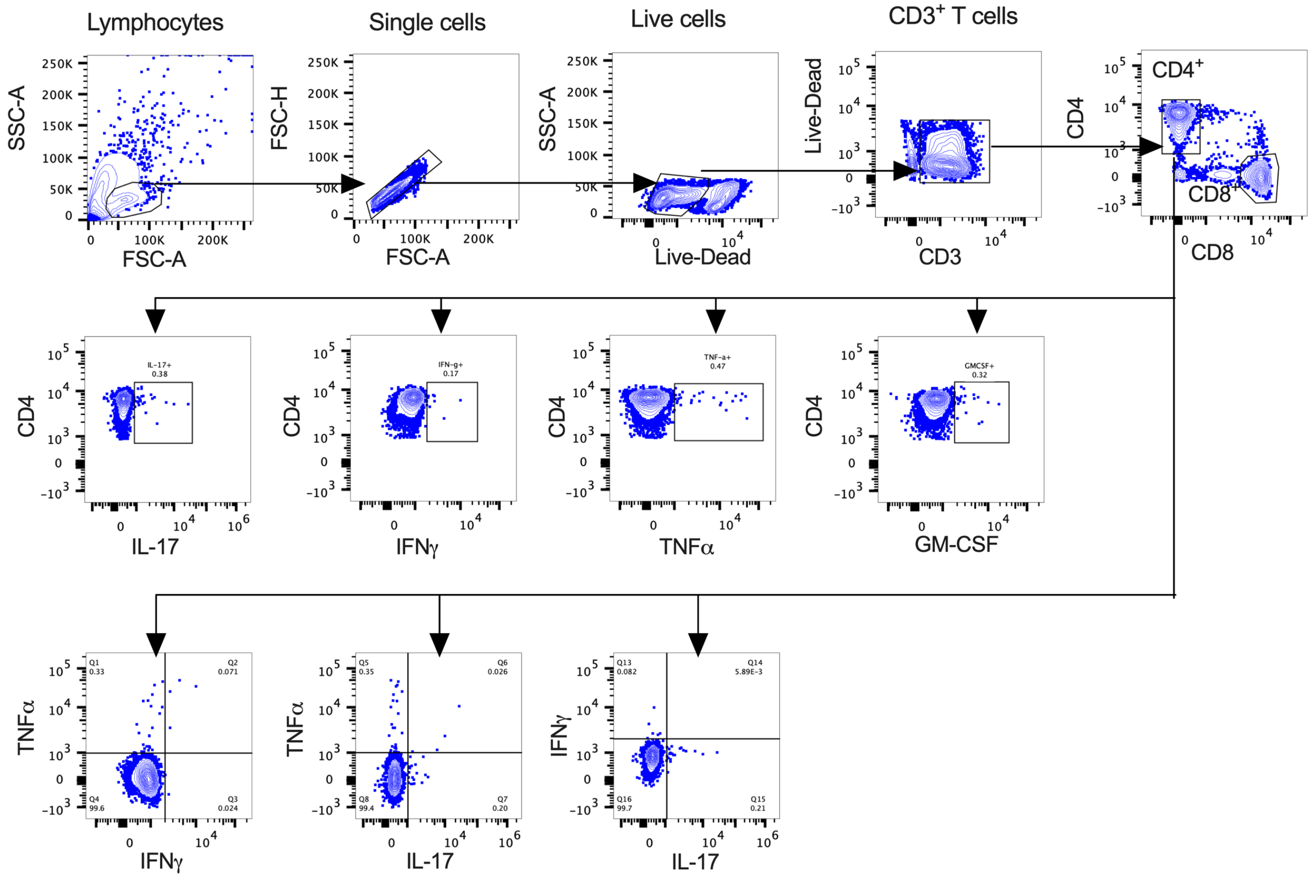

Supplementary Figure 1: Identification of *Mtb*-specific CD4$^+$ T cells. Gating strategy to detect cytokine-producing CD4$^+$ T cells after stimulation with distinct *Mtb* antigens. The shown strategy is for unstimulated PBMCs; the magnitude of *Mtb*-specific cytokine is reported after subtraction of the unstimulated background staining.

Supplementary figure 2:

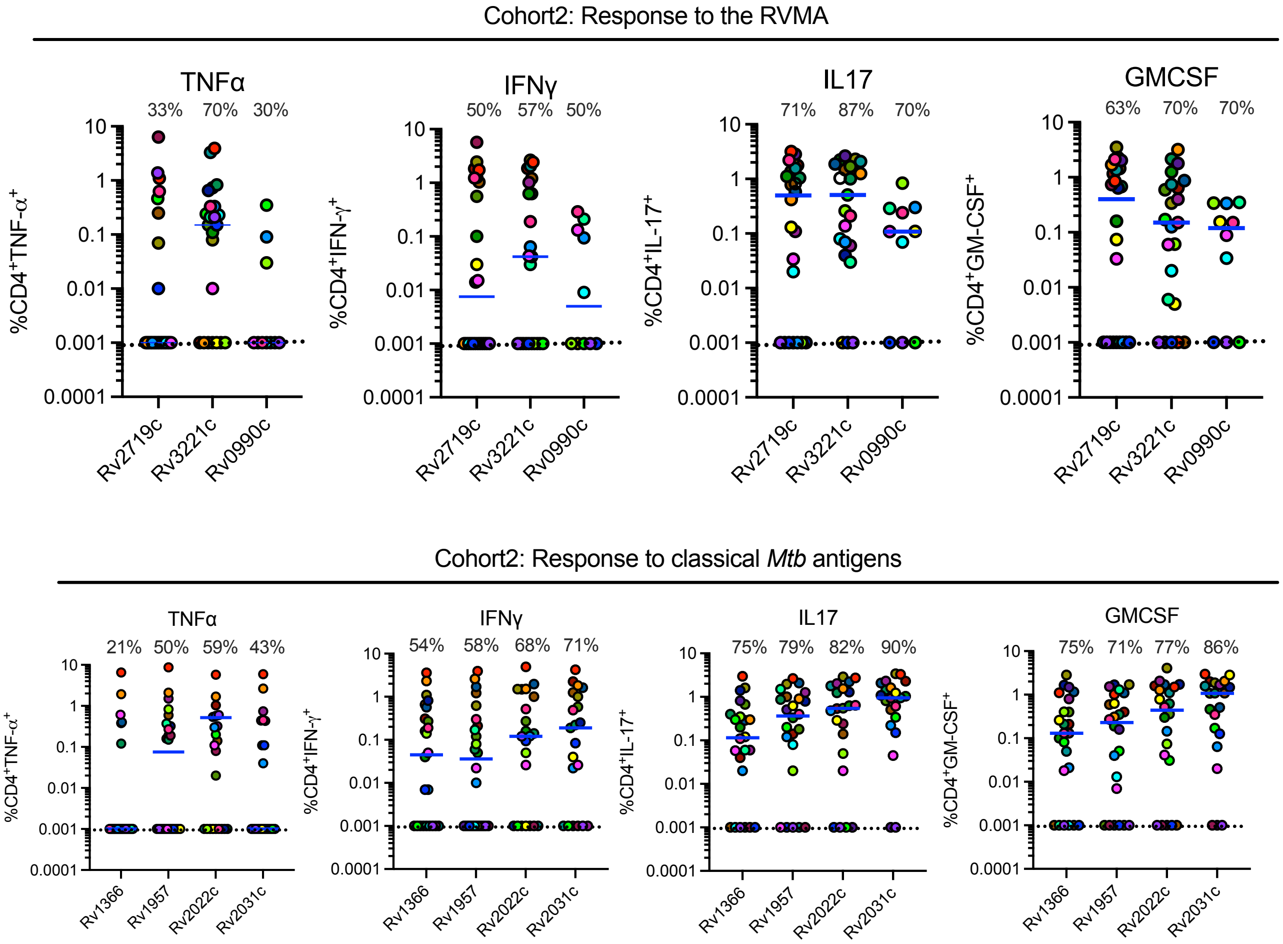

Supplementary Figure 2: Distinct *Mtb* antigens elicit T cell responses with different functional properties (Cohort 2). Procedures and analyses were as described in Figure 1; the samples were obtained from participants in Cohort 2 (AHRI, Addis Ababa, Ethiopia). Results for RVMA (Rv0990c, Rv2719c, and Rv3221c) are shown in the top panel; results for LICA (Rv1366, Rv1957, Rv2022c, and Rv2031c) are shown in the bottom panel.

Supplementary figure 3:

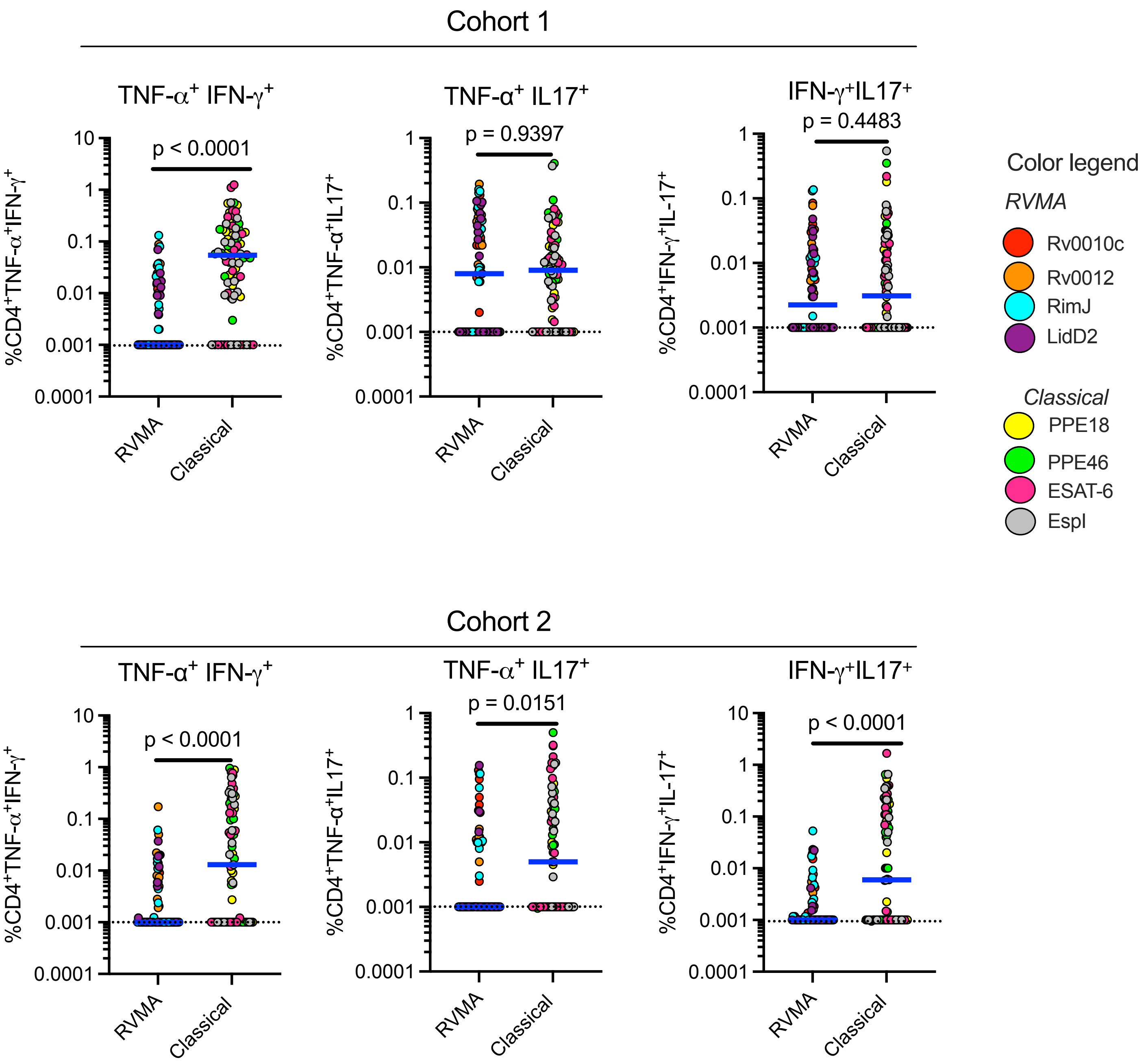

Supplementary Figure 3: The RVMA induce significantly fewer bifunctional CD4$^+$ T cells. Cryopreserved PBMCs from participants in both cohorts were stimulated with distinct antigens (2 µg/ml) for a total of 20 hours in the presence of Golgi Stop and Golgi Plug and costimulatory antibodies anti-CD28 and anti-CD49d and dual cytokine production by CD4$^+$ T cells determined by intracellular cytokine staining. Each color code is for a distinct antigen as indicated; blue line indicates the median cytokine response. Statistics: Mann-Whitney test.

Supplemental Table 1. Demographic and clinical characteristics of study cohorts

**Characteristic**	**Cohort 1**	**Cohort 2**	**p***
Age, Median (Interquartile range), Y	31.5 (20, 49.5)	32.9 (26.7, 38.6)	0.6613
Sex, n (%)			
Male	15 (42%)	24 (56%)	p = 0.2611, Fisher's exact test)
Female	21 (58%)	19 (44%)	
BMI; Median (Interquartile range)	21.95 (20.18, 26.58)	21.80 (19.8, 24.7)	0.8852
HbA1c, %; Median (Interquartile range)	5.5 (5.2,5.7)	5.3 (5, 6.1)	0.7467
QFT Results, IU/mL; Median (Interquartile range)			
TB antigen minus Nil	9.03 (1.95, 10)	5.56 (2.2, 8.23)	**0.0183**
Mitogen minus Nil	5.19 (2.09, 9.46)	8.78 (7.75, 9.72)	**0.0011**
*p = Absolute p, Mann-Whitney unless otherwise specified.			

Supplemental Table 2. Cohort 2: Frequencies of CD4 T cell cytokine responses, by individual antigens

	**RVMA**				**IFNγ-dominant Classical**			
	Rv0010c n=17	Rv0012 n=24	RimJ n=29	LldD2 n=13	PPE18 n=21	PPE46 n=17	ESAT-6 n=21	EspI n=14
TNF	29	58	52	15	52	71	86	86
IFNγ	35	58	55	38	71	82	86	93
IL-17	59	58	62	54	67	88	86	86
GM-CSF	59	79	45	62	57	82	71	79
The values shown reflect the percent of participants whose samples yielded detectable responses, defined as >0.001% of CD4 T cells after stimulation with the indicated antigens.								

Supplemental Table 3. Cohort 2: Frequencies of responders: individual cytokines vs antigen class

	Median % (interquartile range) n = 4 antigens per category		Absolute p (Mann-Whitney)
	**RVMA**	**IFNγ-dominant Classical**	
TNF	40 (19, 56)	78 (57, 85)	0.0571
IFNγ	51 (38, 57)	84 (74, 90)	0.0286
IL-17	58 (54, 61)	85 (71, 87)	0.0286
GM-CSF	60 (48, 75)	74 (61, 78)	0.6857

Supplemental Table 4. Cohort 2: Magnitudes of individual cytokine responses (% of CD4 T cells) vs antigen class

	Median % (interquartile range) n = 4 antigens per category		Absolute p (Mann-Whitney)
	**RVMA**	**IFNγ-dominant Classical**	
TNF	0.001 (0.001, 0.12)	0.35 (0.001, 0.8)	<0.0001
IFNγ	0.001 (0.001, 0.06)	0.3(0.05, 0.72)	<0.0001
IL-17	0.04 (0.001, 0.24)	0.29 (0.09, 0.66)	<0.0001
GM-CSF	0.03 (0.001, 0.22)	0.13 (0.001, 0.32)	0.0662

Values shown for each cytokine and each antigen are median % of all CD4 T cells that express the specified cytokine. For statistical analyses, assays that yielded undetectable levels of the stated cytokine were assigned a value of 0.001%.

Supplemental Table 5. Cohort 2: IL-17 vs IFNγ responses to individual RVMA

	Response Frequencies (% of participants with detectable cytokine+ CD4 T cells)		Response Magnitudes (% of CD4 T cells that are cytokine+) Median (interquartile range)		
	**IL-17**	**IFNγ**	**IL-17**	**IFNγ**	p*
Rv0010c	58	35	0.02 (0.001, 0.115)	0.001 (0.001, 0.047)	0.2437
Rv0012	58	58	0.07 (0.001, 0.385)	0.012 (0.001, 0.0775)	0.0054
RimJ	62	55	0.06 (0.001, 0.32)	0.01 (0.001, 0.087)	0.2157
LldD2	63	46	0.03 (0.001, 0.255)	0.001 (0.001,0.0305)	0.0391
Rv0990c	70	50	0.11 (0.001, 0.29)	0.005 (0.001, 0.15)	0.0781
Rv2719c	71	50	0.5 (0.001, 1.51)	0.008 (0.001, 1.18)	0.1269
Rv3221c	87	57	0.51 (0.06, 1.68)	0.042 (0.001, 1.01)	0.0602
*p values for the comparison of IL-17 vs IFNγ magnitudes (Wilcoxon matched pairs)					

Supplemental Table 6. Cohort 2: Magnitudes of individual cytokine responses (% of CD4 T cells) vs antigen class

	Median % (interquartile range) n = 3 RVMA and n = 4 LICA		Absolute p (Wilcoxon matched pairs)
	**IL-17**	**IFNγ**	
RVMA	0.26 (0.025, 1.35)	0.01 (0.001, 0.63)	0.0059
LICA	0.4 (0.05, 1.24)	0.08 (0.001, 0.58)	0.0003
Values shown for each cytokine and each antigen class are median % of all CD4 T cells that express the specified cytokine. For statistical analyses, assays that yielded undetectable levels of the stated cytokine were assigned a value of 0.001%.			

Tuberculosis Research Unit – Role of Antigen Specific T Cell Responses in the Control of TB (TBRU- ASTRa) Consortium Study members:

**Name**	**Affiliation**
Rafi Ahmed	Emory Vaccine Center, Emory University, Atlanta, GA, USA
Lance Waller	Department of Biostatistics and Bioinformatics, Rollins School of Public Health, Emory University, Atlanta, GA USA
Lisa Elon	Department of Biostatistics and Bioinformatics, Rollins School of Public Health, Emory University, Atlanta, GA USA
Andrea Knezevic	Department of Biostatistics and Bioinformatics, Rollins School of Public Health, Emory University, Atlanta, GA USA
Shirin Jabbarzadeh	Department of Biostatistics and Bioinformatics, Rollins School of Public Health, Emory University, Atlanta, GA USA
Azhar Nizam	Department of Biostatistics and Bioinformatics, Rollins School of Public Health, Emory University, Atlanta, GA USA
Hao Wu	Department of Biostatistics, Rollins School of Public Health, Emory University, Atlanta, GA 30322
Seegar Swanson	Department of Biostatistics, Rollins School of Public Health, Emory University, Atlanta, GA 30322
Yunyun Chen	Department of Biostatistics, Rollins School of Public Health, Emory University, Atlanta, GA 30322
Wendy Whatney	Emory Vaccine Center, Emory University, Atlanta, GA, USA
Melanie Quezada	Emory Vaccine Center, Emory University, Atlanta, GA, USA
Loren Sasser	Emory Vaccine Center, Emory University, Atlanta, GA, USA
Ranjna Madan Lala	Emory Vaccine Center, Emory University, Atlanta, GA, USA
Tawania Fergus	Department of Medicine, New York University School of Medicine, New York, NY, USA. Present address: Division of Rheumatology, Albert Einstein College of Medicine, Bronx, NY
Toidi Adekambi	Emory Vaccine Center, Emory University School of Medicine
Deepak Kaushal	Texas Biomedical Research Institute, San Antonio, TX, USA
Nadia Golden	Tulane National Primate Research Center, Tulane University School of Medicine, Covington, LA, USA
Taylor Foreman	Tulane National Primate Research Center, Tulane University School of Medicine, Covington, LA, USA
Allison Bucsan	Tulane National Primate Research Center, Tulane University School of Medicine, Covington, LA, USA. Present address: Vaccine Research Center, National Institute of Allergy and Infectious Diseases
Chris Ibegbu	Emory Vaccine Center, Emory University, Atlanta, GA, USA
Susanna Contraras Alcantra	Emory Vaccine Center, Emory University, Atlanta, GA, USA

Alessandro Sette	Center for Vaccine Innovation, La Jolla Institute for Immunology, La Jolla, CA, USA
Salim Allana	Department of Epidemiology, Emory University Rollins School of Public Health, Atlanta, GA, USA
Angela Campbell	Department of Epidemiology, Emory University Rollins School of Public Health, Atlanta, GA, USA
Sarita Shah	Department of Epidemiology, Emory University Rollins School of Public Health, Atlanta, GA, USA
Susan Ray	Division of Infectious Diseases, Department of Medicine, Emory University School of Medicine, Atlanta, Georgia, USA.
James Brust	Division of Infectious Diseases, Department of Medicine, Albert Einstein College of Medicine and Montefiore Medical Center, Bronx, New York, USA.
Jeffrey M. Collins	Division of Infectious Diseases, Department of Medicine, Emory University School of Medicine, Atlanta, Georgia, USA.
Meghan Franczek	Department of Epidemiology, Emory University Rollins School of Public Health, Atlanta, GA, USA
Jenna Daniel	Department of Epidemiology, Emory University Rollins School of Public Health, Atlanta, GA, USA
Alison GC Smith	Department of Epidemiology, Emory University Rollins School of Public Health, Atlanta, GA, USA
Anirudh Rao	Department of Epidemiology, Rollins School of Public Health, Emory University
Rebecca Goldstein	Department of Epidemiology, Rollins School of Public Health, Emory University
Madeleine Kabongo	Department of Epidemiology, Emory University Rollins School of Public Health, Atlanta, GA, USA
Alawode Oladele	DeKalb County Board of Health, Atlanta, GA, USA
Janet Agaya	Center for Global Health Research, Kenya Medical Research Institute, Kisumu, Kenya
Jeremiah Khayumbi	Center for Global Health Research, Kenya Medical Research Institute, Kisumu, Kenya
Joan Tonui	Center for Global Health Research, Kenya Medical Research Institute, Kisumu, Kenya
Benson Muchiri	Center for Global Health Research, Kenya Medical Research Institute, Kisumu, Kenya
Joshua Ongalo	Center for Global Health Research, Kenya Medical Research Institute, Kisumu, Kenya
Dickson Gethi	Center for Global Health Research, Kenya Medical Research Institute, Kisumu, Kenya
Felix Hayara Odhiambo	Center for Global Health Research, Kenya Medical Research Institute, Kisumu, Kenya
Dorine Awilly	Center for Global Health Research, Kenya Medical Research Institute, Kisumu, Kenya
Albert Ochieng Okumu	Center for Global Health Research, Kenya Medical Research Institute, Kisumu, Kenya

Abraham Aseffa	Mycobacterial Disease Research Directorate, Armauer Hansen Research Institute, Addis Ababa, Ethiopia
Medina Hamza	Mycobacterial Disease Research Directorate, Armauer Hansen Research Institute, Addis Ababa, Ethiopia
Yonas Abebe	Mycobacterial Disease Research Directorate, Armauer Hansen Research Institute, Addis Ababa, Ethiopia
Fisseha Mulate	Mycobacterial Disease Research Directorate, Armauer Hansen Research Institute, Addis Ababa, Ethiopia
Mekdelawit Wondiyfraw	Mycobacterial Disease Research Directorate, Armauer Hansen Research Institute, Addis Ababa, Ethiopia
Firaol Degaga	Mycobacterial Disease Research Directorate, Armauer Hansen Research Institute, Addis Ababa, Ethiopia
Daniel Getachew	Mycobacterial Disease Research Directorate, Armauer Hansen Research Institute, Addis Ababa, Ethiopia
Dawit Tayachew Bere	Mycobacterial Disease Research Directorate, Armauer Hansen Research Institute, Addis Ababa, Ethiopia
Meaza Zewdu	Mycobacterial Disease Research Directorate, Armauer Hansen Research Institute, Addis Ababa, Ethiopia
Daniel Mussa	Mycobacterial Disease Research Directorate, Armauer Hansen Research Institute, Addis Ababa, Ethiopia
Bezalam Tesfaye	Mycobacterial Disease Research Directorate, Armauer Hansen Research Institute, Addis Ababa, Ethiopia
Selam Jemberu	Mycobacterial Disease Research Directorate, Armauer Hansen Research Institute, Addis Ababa, Ethiopia
Azeb Tarekegn	Mycobacterial Disease Research Directorate, Armauer Hansen Research Institute, Addis Ababa, Ethiopia
Gebeyehu Assefa	Mycobacterial Disease Research Directorate, Armauer Hansen Research Institute, Addis Ababa, Ethiopia
Gutema Jebessa	Mycobacterial Disease Research Directorate, Armauer Hansen Research Institute, Addis Ababa, Ethiopia
Zewdu Solomon	Mycobacterial Disease Research Directorate, Armauer Hansen Research Institute, Addis Ababa, Ethiopia
Sebsibe Neway	Mycobacterial Disease Research Directorate, Armauer Hansen Research Institute, Addis Ababa, Ethiopia
Jemal Hussein	Mycobacterial Disease Research Directorate, Armauer Hansen Research Institute, Addis Ababa, Ethiopia
Tsegaye Hailu	Mycobacterial Disease Research Directorate, Armauer Hansen Research Institute, Addis Ababa, Ethiopia
Alemayehu Geletu	Mycobacterial Disease Research Directorate, Armauer Hansen Research Institute, Addis Ababa, Ethiopia
Edom Girma	Mycobacterial Disease Research Directorate, Armauer Hansen Research Institute, Addis Ababa, Ethiopia
Million Legesse	Mycobacterial Disease Research Directorate, Armauer Hansen Research Institute, Addis Ababa, Ethiopia
Mitin Wendaferew	Mycobacterial Disease Research Directorate, Armauer Hansen Research Institute, Addis Ababa, Ethiopia

Hirut Solomon	Mycobacterial Disease Research Directorate, Armauer Hansen Research Institute, Addis Ababa, Ethiopia
Zenebech Assefa	Mycobacterial Disease Research Directorate, Armauer Hansen Research Institute, Addis Ababa, Ethiopia
Mahlet Mekuria	Mycobacterial Disease Research Directorate, Armauer Hansen Research Institute, Addis Ababa, Ethiopia
Misker Kedir	Mycobacterial Disease Research Directorate, Armauer Hansen Research Institute, Addis Ababa, Ethiopia
Eleni Zeleke	Mycobacterial Disease Research Directorate, Armauer Hansen Research Institute, Addis Ababa, Ethiopia
Rediet Zerihun	Mycobacterial Disease Research Directorate, Armauer Hansen Research Institute, Addis Ababa, Ethiopia
Selam Dechasa	Mycobacterial Disease Research Directorate, Armauer Hansen Research Institute, Addis Ababa, Ethiopia
Emebet Haile	Mycobacterial Disease Research Directorate, Armauer Hansen Research Institute, Addis Ababa, Ethiopia
Nahom Getachew	Mycobacterial Disease Research Directorate, Armauer Hansen Research Institute, Addis Ababa, Ethiopia
Firaol Wagari	Mycobacterial Disease Research Directorate, Armauer Hansen Research Institute, Addis Ababa, Ethiopia
Ruth Mekonnen	Mycobacterial Disease Research Directorate, Armauer Hansen Research Institute, Addis Ababa, Ethiopia
Samuel Bayu	Mycobacterial Disease Research Directorate, Armauer Hansen Research Institute, Addis Ababa, Ethiopia
Melat Gebre-Medhin	Mycobacterial Disease Research Directorate, Armauer Hansen Research Institute, Addis Ababa, Ethiopia
Alemayehu Kifle	Mycobacterial Disease Research Directorate, Armauer Hansen Research Institute, Addis Ababa, Ethiopia
